# Mesenchymal Stem Cells as Anti-Inflammatory Agents in Chronic Kidney Disease: A Systematic Review and Meta-Analysis

**DOI:** 10.3390/cells14171313

**Published:** 2025-08-24

**Authors:** Lukman Pura, Raeni Dwi Putri, Muh. Arya Prahmana, Muhammad Palar Wijaya, Ria Bandiara, Ahmad Faried, Rudi Supriyadi

**Affiliations:** 1 Doctoral Study Program of Medical Sciences, Faculty of Medicine, Universitas Padjadjaran, Bandung 40132, Indonesia; 2 Dr. H. Abdul Moeloek Regional General Hospital, Bandar Lampung 35112, Indonesia; 3 Master Study Program of Biomedical Sciences, Faculty of Medicine, Universitas Padjadjaran, Bandung 40132, Indonesia; 4 Division of Nephrology and Hypertension, Department of Internal Medicine, Faculty of Medicine, Universitas Padjadjaran/Dr. Hasan Sadikin General Hospital, Bandung 40161, Indonesia; 5 Department of Neurosurgery and Stem Cell Working Group, Faculty of Medicine, Universitas Padjadjaran/Padjadjaran University Hospital, Jatinangor 45363, Indonesia

**Keywords:** mesenchymal stem cells, chronic kidney disease, kidney failure, inflammation, stem cell transplantation

## Abstract

Background: Chronic kidney disease (CKD) is largely driven by inflammation. Mesenchymal stem cells (MSCs) show therapeutic potential; however, their efficacy across CKD etiologies remains unclear. Methods: Comprehensive searches were conducted in PubMed, Cochrane, ScienceDirect, Scopus and Google Scholar. Effect sizes for inflammation and renal function outcomes were meta-analyzed. Results: Of 2514 studies screened, 52 met inclusion criteria (49 animal studies, 3 randomized controlled trials). In animal models, MSCs significantly reduced interleukin-6 (mean difference [MD] = −155.80; 95% CI: −249.10, −62.51; *p* = 0.001) and tumor necrosis factor-α (TNF-α) (MD = −35.53; 95% CI: −52.75, −18.30; *p* < 0.0001). In patients, TNF-α reduction was not significant (MD = −0.74; 95% CI: −2.20, 0.73; *p* = 0.32). Serum creatinine decreased in animals (MD = −0.38; 95% CI: −0.46, −0.29; *p* < 0.00001), but not in patients (MD = −0.59; 95% CI: −1.92, 0.74; *p* = 0.39). Blood urea nitrogen decreased in animals (MD = −19.27; 95% CI: −23.50, −15.04; *p* < 0.00001), and glomerular filtration rate improved (standardized MD = 1.83; 95% CI: 0.51, 3.15; *p* = 0.007), with no change in patients. Conclusion: MSCs improve inflammation and renal function in CKD animal models; however, evidence in patients remains inconclusive.

## 1. Introduction

Chronic kidney disease (CKD), one of the most recognized public health problems, affects approximately 10% of the global population. It is a degenerative condition that often leads to end-stage renal failure, placing a considerable financial burden on healthcare systems [1,2].

Persistent inflammation plays a central role in CKD progression by promoting a profibrotic microenvironment through elevated levels of cytokines, such as tumor necrosis factor-alpha (TNF-α), interleukin-6 (IL-6), and reactive oxygen species (ROS). [3,4] These inflammatory pathways accelerate CKD progression, rendering inflammation a critical therapeutic target [5,6].

Current CKD therapies, such as angiotensin-converting enzyme (ACE) inhibitors, angiotensin II receptor blockers, and anti-inflammatory agents, focus on controlling comorbid conditions, such as hypertension, cardiovascular disease, and diabetes. However, they remain insufficient to halt disease progression, highlighting the need for novel therapeutic approaches [7]. Mesenchymal stem cells (MSCs), with anti-inflammatory, antioxidative, and regenerative properties, are a promising therapeutic alternative. MSCs release growth factors and cytokines that influence parenchymal cells and promote tissue regeneration [8,9]. Preclinical studies have shown that MSCs modulate oxidative stress, reduce inflammation, and prevent kidney damage in animal models. This has renewed interest in their potential for CKD therapy [10,11].

Despite encouraging animal data, clinical trials have shown mixed results: some have reported significant improvements in renal function, whereas others have shown limited or no benefits. These discrepancies likely arise from heterogeneity in MSC sources, dosages, administration methods, and patient populations. Previous systematic reviews have largely concentrated on diabetic nephropathy or specific MSC sources, thereby limiting generalizability. To date, no meta-analysis has systematically evaluated the anti-inflammatory and renoprotective potential of MSC therapy across diverse CKD etiologies and induction methods. This review addresses this gap by evaluating MSC therapy across preclinical and clinical studies involving various causes of CKD, focusing on key inflammatory and fibrotic mediators—including IL-6, TNF-α, and TGF-β—as well as functional markers like creatinine, BUN, GFR, and urinary protein indices. This review aims to systematically assess whether MSC exerts anti-inflammatory and renoprotective effects across diverse CKD models and patient populations. Particular attention is given to how treatment characteristics—such as MSC source, delivery method, and follow-up duration—may influence therapeutic outcomes.

## 2. Methods

### 2.1. Data Sources and Search Strategy

This systematic review and meta-analysis followed the Cochrane Guidelines for Systematic Reviews of Interventions [12]. It was registered with PROSPERO (CRID: CRD42024587614) on 16 September 2024, and adheres to the Preferred Reporting Items for Systematic Reviews and Meta-Analyses (PRISMA) [13]. The primary research question examined MSC therapy’s effect on inflammation in CKD compared to controls. Comprehensive searches were conducted in PubMed, Cochrane CENTRAL, ScienceDirect, and Scopus for English-language publications, focusing on randomized controlled trials (RCTs), non-RCTs with comparator groups, and animal studies through 7 September 2024. In addition to database searches, a gray literature search was conducted using Google Scholar. We used combinations of terms such as “mesenchymal stem cells,” “chronic kidney disease,” “inflammation,” and “renal function”. The search strategy was independently developed and tested by three authors (LP, RDP, and MAP), and any discrepancies in search terms, Boolean combinations, or database syntax were resolved through consensus discussions and iterative refinement. Finalized search strategy for all sources, including gray literature, are provided in Supplementary Table S1.

### 2.2. Eligibility Criteria

Studies were eligible if they met the following criteria: original research articles (RCTs and non-RCTs) involving MSC therapy in patients with CKD or animal models of CKD, with a comparator group. To avoid limiting the analysis to a small number of available human RCTs, we included both animal and human studies in this review to provide a more comprehensive evaluation of the therapeutic potential of MSCs in CKD. The study population included adults (≥18 years) with CKD of any etiology and animal models of CKD induced through surgical, chemical, or genetic methods. MSCs could be derived from any source, including bone marrow, adipose tissue, and umbilical cord. We excluded abstracts, conference proceedings, systematic reviews, correspondence, case studies, and studies published in languages other than English.

### 2.3. Study Selection

**Figure 1:**
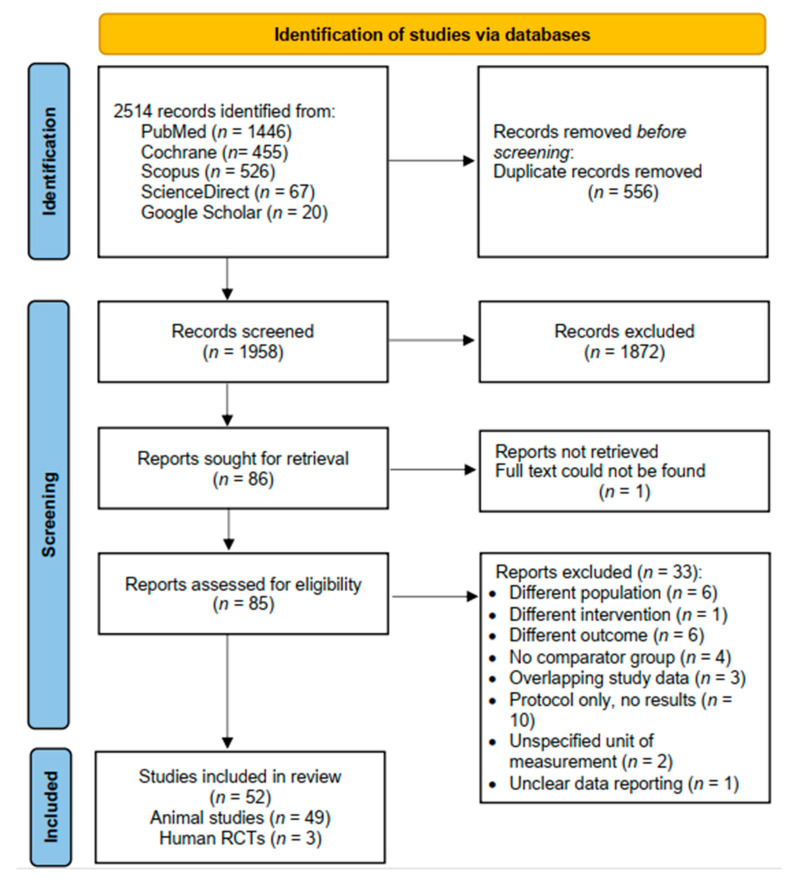
PRISMA flow diagram of study screening and selection process. Figure created using the PRISMA 2020 flow diagram generator (http://www.prisma-statement.org/, accessed on 21 September 2024), based on the PRISMA 2020 statement.

In addition to the database searches, the reference lists of the included studies were manually examined. Duplicate citations were eliminated, and the titles and abstracts of the retrieved articles were independently reviewed by RDP and MAP, and any differences were resolved through discussion with MPW. The complete texts of the remaining articles were thoroughly analyzed. The screening process is summarized in the PRISMA flowchart, presented in Figure 1.

### 2.4. Data Extraction

Four authors independently performed the data extraction (LP, RDP, MAP, and MPW). Discrepancies were addressed by a fifth author (RB). Data extracted included the study author, publication year, study design, sample size, intervention and control group details, source of MSC, follow-up duration, and outcomes, including inflammatory markers, tubular injury marker, and renal function measures (serum creatinine [SCr], blood urea nitrogen [BUN], glomerular filtration rate [GFR], proteinuria, albuminuria, protein/creatinine ratio [PCR], and albumin/creatinine ratio [ACR]). The extracted data were cross-verified and reviewed by all five authors. To address variability across studies, continuous outcomes were standardized using several strategies. Units were converted to a common scale where applicable (e.g., mg/dL for creatinine, pg/mL for cytokines). When standard deviations (SDs) were not reported, they were estimated from standard errors and confidence intervals using established formulas. If numeric values were not reported but data were presented graphically (e.g., bar graphs, line plots), values were extracted using digital measurement of chart elements (e.g., axis scales and bar heights). If means and variability could not be reliably derived, the study was excluded from quantitative synthesis but retained in the review. Supplementary figures, tables, and text were cross-checked for completeness.

### 2.5. Risk-of-Bias Assessment

We used the RoB-2 tool for RCTs to assess the risk of bias, evaluating domains such as randomization, deviations from interventions, missing outcome data, outcome measurement, and reporting bias [14], and the SYRCLE’s Risk of Bias tool was used for animal studies, considering selection, performance, detection, attrition, and reporting bias [15]. Three authors independently performed risk-of-bias assessment (RB, AF, RS).

### 2.6. Data Synthesis and Statistical Analysis

Continuous outcomes were analyzed using mean differences (MDs) or standardized MDs (SMDs) with 95% confidence intervals (CIs) using RevMan 5.4 software. Outcomes of interest included inflammatory markers: interleukin 6 (IL-6), tumor necrosis alpha (TNF-α), transforming growth factor beta (TGF- β), and nuclear factor kappa-light-chain-enhancer of activated B cells (NF-κB); tubular injury marker: kidney injury molecule-1 (KIM-1); and renal function indicators: SCr, BUN, GFR, proteinuria, albuminuria, urine PCR, and urine ACR. Heterogeneity was assessed using the I^2^ statistic, and publication bias was assessed using funnel plots. A random-effects model was used for most meta-analyses, based on anticipated clinical and methodological diversity across studies, as well as high heterogeneity level based on the I2 statistic. Clinical heterogeneity was expected due to variability in CKD etiology (e.g., diabetic nephropathy, nephrectomy, chemical-induced nephropathy), MSC sources (e.g., bone marrow, umbilical cord, adipose), delivery methods, and follow-up durations. Methodological heterogeneity was also present across outcome measurement techniques (e.g., ELISA, PCR, Western blot). Recognizing the substantial methodological differences between animal studies and human RCTs, including species differences, MSC delivery approaches, and outcome measurement techniques, we conducted separate meta-analyses for each study design. Subgroup analyses were also carried out, based on study design (RCTs and animal studies), follow-up duration (0 to ≤2 weeks; >2 to ≤4 weeks; >4 to ≤8 weeks; >8 to ≤12 weeks; and >12 weeks), CKD model or type of CKD induction, source of MSC, and MSC-derived product. The subgroup analyses were prespecified to address anticipated sources of heterogeneity. MSC source was considered, due to known differences in immunomodulatory potential and cytokine profiles across tissues. Follow-up duration was included to assess whether therapeutic effects were transient or sustained over time. Study design (animals vs. humans) was analyzed separately, to account for differences in disease stage, biological variability, and study conditions. CKD induction method was evaluated because MSC efficacy may vary, depending on whether the model reflects diabetic, toxic, ischemic, or obstructive pathology. Finally, MSC-derived products (whole cells vs. exosomes) were analyzed independently, given their differing mechanisms of action and pharmacokinetics.

Trial Sequential Analysis (TSA) was performed for outcomes evaluated in human RCTs (serum creatinine, TNF-α, GFR, and ACR) using TSA software (Copenhagen Trial Unit). Required information size (RIS) was calculated with conventional and alpha-spending boundaries. TSA was conducted to assess the conclusiveness of the available evidence and to control both type I and type II errors, ensuring adequate statistical power and preventing premature conclusions.

Publication bias was assessed for outcomes that included more than 10 individual studies. We evaluated publication bias using a combination of visual inspection of funnel plots and the regression-based Egger’s test for small-study effects. Funnel plot asymmetry was examined qualitatively, and Egger’s test was conducted to statistically assess small-study effects, with a *p*-value < 0.05 considered indicative of potential publication bias. In addition, we assessed the certainty of evidence for key outcomes using the GRADE (Grading of Recommendations, Assessment, Development and Evaluation) approach, considering risk of bias, inconsistency, indirectness, imprecision, and publication bias, and summarized findings in a Summary of Findings table.

## 3. Results

### 3.1. Characteristics of Studies

The screening process is summarized in the PRISMA flowchart, presented in Figure 1. Our initial search yielded 2514 records, of which 556 were duplicates. After screening the titles and abstracts, 1872 articles were deemed irrelevant and were excluded. This left 86 articles for retrieval, with one full-text article not retrieved, leading to 85 articles for a detailed review. After applying the inclusion and exclusion criteria, 33 studies were excluded. The exclusion criteria were as follows: different populations (*n* = 6), different interventions (*n* = 1), different outcomes (*n* = 6), absence of a comparator group (*n* = 4), overlapping study data (*n* = 3), protocol only, with no results (*n* = 10), unspecified units of measurement (*n* = 2), and unclear data reporting (*n* = 1). Details of the 33 studies excluded after full-text screening, including study title, authors, and specific reasons for exclusion, are provided in Supplementary Table S2.

Finally, 52 studies were included, with 49 based on animals [11,16,17,18,19,20,21,22,23,24,25,26,27,28,29,30,31,32,33,34,35,36,37,38,39,40,41,42,43,44,45,46,47,48,49,50,51,52,53,54,55,56,57,58,59,60,61,62,63] and 3 on human RCTs [64,65,66]. Among the 49 animal studies, 41 used rat models, 4 used mouse models, 2 used rhesus macaque models, 1 used the treeshrew model, and 1 used the rabbit model. CKD was induced using various methods, including streptozotocin (STZ) injection to induce diabetic nephropathy (DN); ureteral unilateral obstruction (UUO, injection of aristolochic acid; genetically modified COL4A3 deficiency to induce renal fibrosis; and adriamycin-, cisplatin-, or adenine-induced nephropathy, as well as nephrectomy.

Although all the animal studies used MSCs, their sources, dosages, administration frequencies, and timing differed. MSCs were derived from the bone marrow in 22 studies, umbilical cord in 14 studies, adipose tissue in 5 studies, amniotic membrane in 3 studies, kidney tissue in 2 studies, pluripotent stem cells in 2 studies, and peripheral blood mononuclear cells in 1 study. The details of these studies are summarized in Table 1.

Three human studies, comprising 86 participants, were RCTs. Two studies recruited patients with DN, whereas the other recruited patients with CKD of any etiology. MSCs were bone-marrow MSCs in two studies [64,65], and umbilical-cord MSCs in one study [66]. All RCTs administered MSCs intravenously at varying dosages, frequencies, and durations (72, 18, and 48 weeks). The details of these studies are presented in Table 2.

### 3.2. Primary Outcomes (Inflammatory Mediators and Tubular Injury Marker)

**Figure 2:**
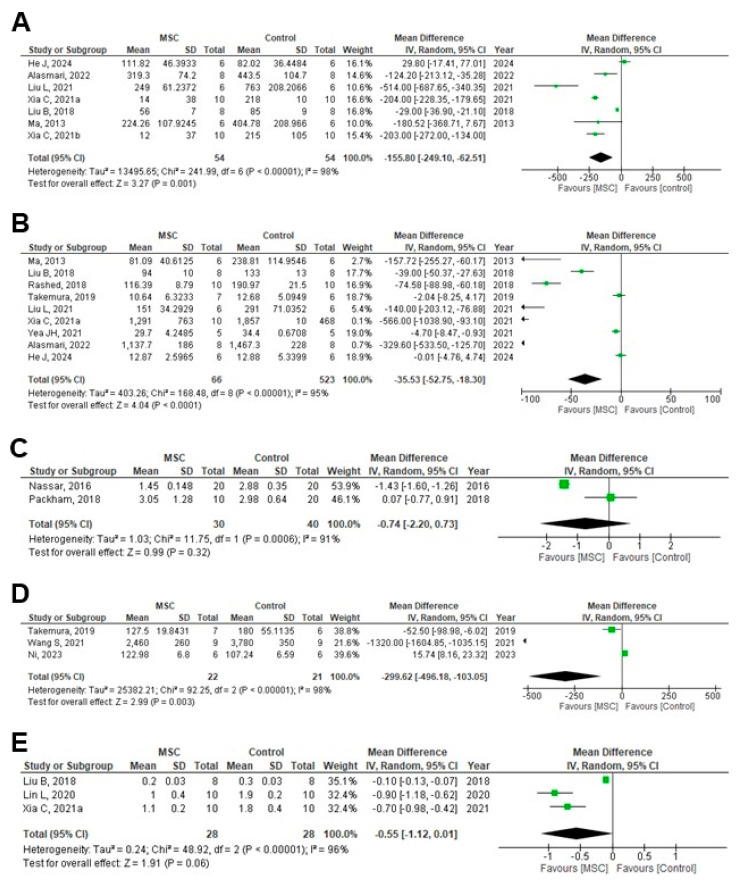
Forest plots of the effect of MSC treatment on inflammatory and oxidative stress mediators: (**A**) Serum IL-6 (pg/mL), (**B**) Serum TNF-α (pg/mL) in animal studies, (**C**) Serum TNF-α (pg/mL) in human RCTs, (**D**) KIM-1 in animal studies, (**E**) Nf-kB in animal studies [11,17,19,22,23,24,27,29,30,31,39,42,53,61,62,63,65,66]. Figures were generated using Review Manager (RevMan) version 5.4, the Cochrane Collaboration.

Serum interleukin (IL-6) was assessed by enzyme-linked immunosorbent assay, showing a significant reduction in the MSC-treated group compared with that in the control group (MD = −155.80; 95% CI: −249.10 to −62.51; *p* = 0.001; I^2^ = 98%) (Figure 2A). Serum TNF-α, measured using enzyme-linked immunosorbent assay, showed significant reductions in the MSC-treated group among animal studies (MD = −35.53; 95% CI: −52.75 to −18.30; *p* < 0.0001; I^2^ = 95%) (Figure 2B), whereas the difference was not significant in patients with CKD enrolled in human RCTs (MD = −0.74; 95% CI: −2.20 to 0.73; *p* = 0.32; I^2^ = 91%) (Figure 2C). TSA confirmed that the sample size for TNF-α in RCTs was inadequate, and no firm conclusion could be drawn (Figure S1). Three animal studies reported KIM-1 and yielded a significant difference (MD = −299.62; 95% CI: −496.18 to −103.05; *p* = 0.003; I^2^ = 98%) (Figure 2D). Another three animal studies reported NF-κB, which showed reductions in the MSC-treated groups that were not statistically significant (MD = −0.55; 95% CI: −1.12 to 0.01; *p* = 0.06; I^2^ = 96%) (Figure 2E).

**Figure 3:**
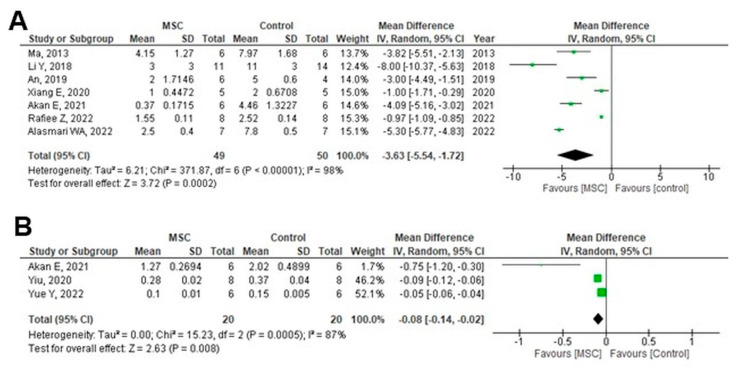
Forest plots of the effect of MSC treatment on renal fibrosis: (**A**) Kidney Tissue TGF-β by measuring mRNA expression in animal studies, (**B**) Kidney Tissue TGF-β by measuring TGF-β/β-actin in animal studies [22,23,24,28,33,36,37,41,53]. Figures were generated using Review Manager (RevMan) version 5.4, the Cochrane Collaboration.

Kidney tissue TGF-β levels were evaluated using various methods, including TGF-β mRNA expression by polymerase chain reaction and relative density or TGF-β/β-actin expression by Western blotting. Significant decreases in TGF-β were observed in the MSC-treated group, both for mRNA expression (MD = −3.63; 95% CI: −5.54 to −1.72; *p* = 0.0002; I^2^ = 98%) (Figure 3A) and TGF-β/β-actin (MD = −0.08; 95% CI: −0.14 to −0.02; *p* = 0.008; I^2^ = 87%) (Figure 3B).

### 3.3. Secondary Outcomes

#### Renal Function (SCr, BUN, and GFR)

**Figure 4:**
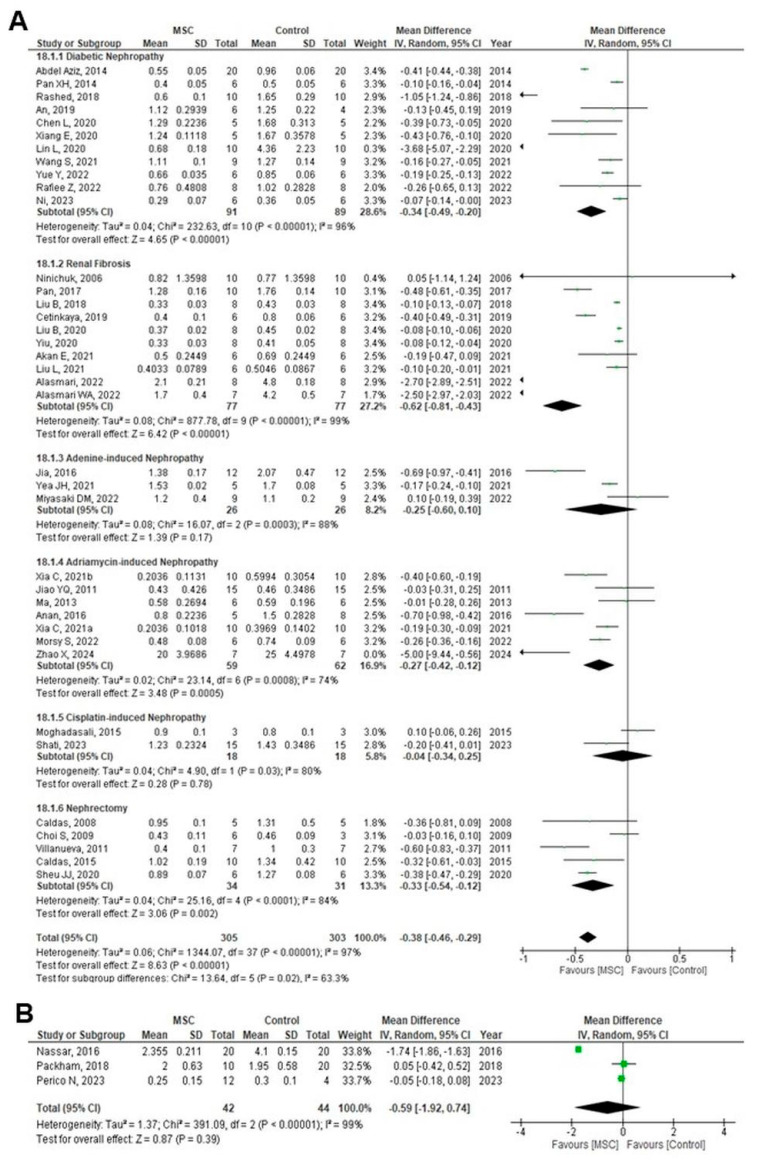
Forest plots of the effect of MSC treatment on (**A**) serum creatinine (mg/dL) in animal studies based on CKD model or type of CKD induction [11,16,19,20,21,22,23,24,27,28,29,31,32,33,34,35,36,37,38,42,43,45,47,50,51,53,54,55,57,58,59,60,62,63], (**B**) serum creatinine (mg/dL) in human RCTs [44,45,46,47,48,49,50,51,52,53,54,55,56,57,58,59,60,61,62,63,64]. Figures were generated using Review Manager (RevMan) version 5.4, the Cochrane Collaboration.

Thirty-eight studies evaluated SCr levels, and a meta-analysis was performed to conduct a subgroup analysis based on CKD models or induction types in animal studies, including DN, renal fibrosis, nephrectomy, and nephropathy induced by adriamycin, adenine, or cisplatin. SCr levels were significantly reduced in the MSC-treated group (MD = −0.38; 95% CI: −0.46 to −0.29; *p* < 0.00001; I^2^ = 63.3%) (Figure 4A).

SCr levels were analyzed in subgroups by follow-up duration after MSC treatment: 0–≤2 weeks, >2–≤4 weeks, >4–≤8 weeks, >8–≤12 weeks, and >12 weeks. Significant reductions in SCr were observed in the MSC group at all time points except > 12 weeks, with an overall effect size of MD = −0.39 (95% CI: −0.47 to −0.30; *p* < 0.00001; I^2^ = 86.3%) (Figure S2). At >12 weeks, this reduction was not significant (MD = −0.06; 95% CI: −0.17 to 0.05; *p* = 0.27; I^2^ = 0%) (Figure S2). Similarly, three human RCTs assessing SCr at >12 weeks found no significant difference between MSC and control groups (MD = −0.59; 95% CI: −1.92 to 0.74; *p* = 0.39; I^2^ = 99%) (Figure 4B). TSA for SCr outcome indicated that the required information size was not reached, and the cumulative Z-curve did not cross conventional or alpha-spending boundaries, suggesting insufficient evidence for a conclusive effect (Figure S3).

Subgroup analysis by MSC source showed significant SCr reductions with MSCs derived from the umbilical cord, bone marrow, amnion, adipose tissue, or other tissues, with reduced heterogeneity (MD = −0.38; 95% CI: −0.46 to −0.29; *p* < 0.0001; I^2^ = 84.7%) (Figure S4). Both whole cells and exosomes significantly reduced SCr levels (MD = −0.39; 95% CI: −0.48 to −0.30; *p* < 0.0001; I^2^ = 78.4%) (Figure S5). Among all subgroup analyses, the CKD induction type showed the lowest heterogeneity, suggesting that it may be a key source of heterogeneity.

**Figure 5:**
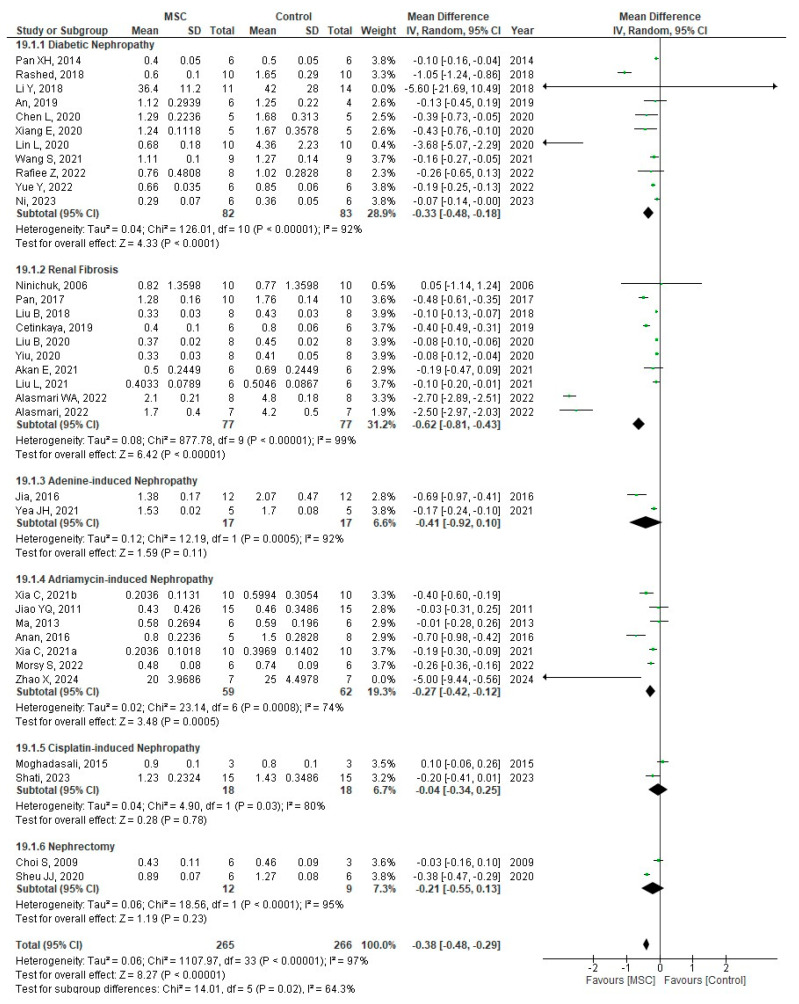
Forest plot of the effect of MSC treatment on BUN (mg/dL) in animal studies based on CKD model or type of CKD induction [11,16,19,20,22,23,24,27,28,29,30,31,32,33,34,35,36,37,38,41,42,43,45,46,47,50,53,55,57,59,61,62,63]. Figures were generated using Review Manager (RevMan) version 5.4, the Cochrane Collaboration.

**Figure 6:**
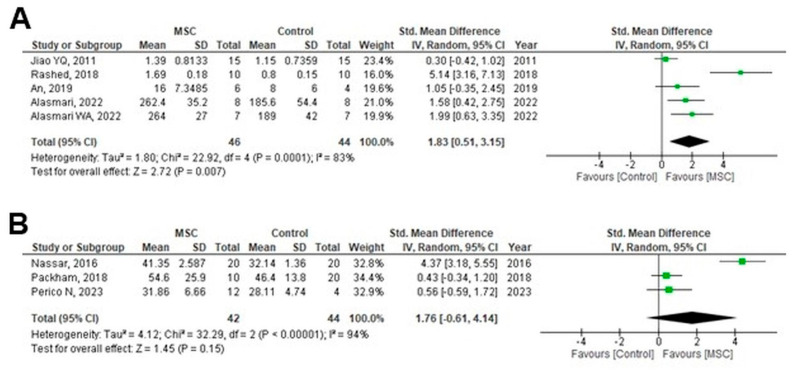
Forest plot of the effect of MSC treatment on GFR in (**A**) animal studies [24,27,37,42,55], (**B**) human RCTs [64,65,66]. Figures were generated using Review Manager (RevMan) version 5.4, the Cochrane Collaboration.

**Figure 7:**
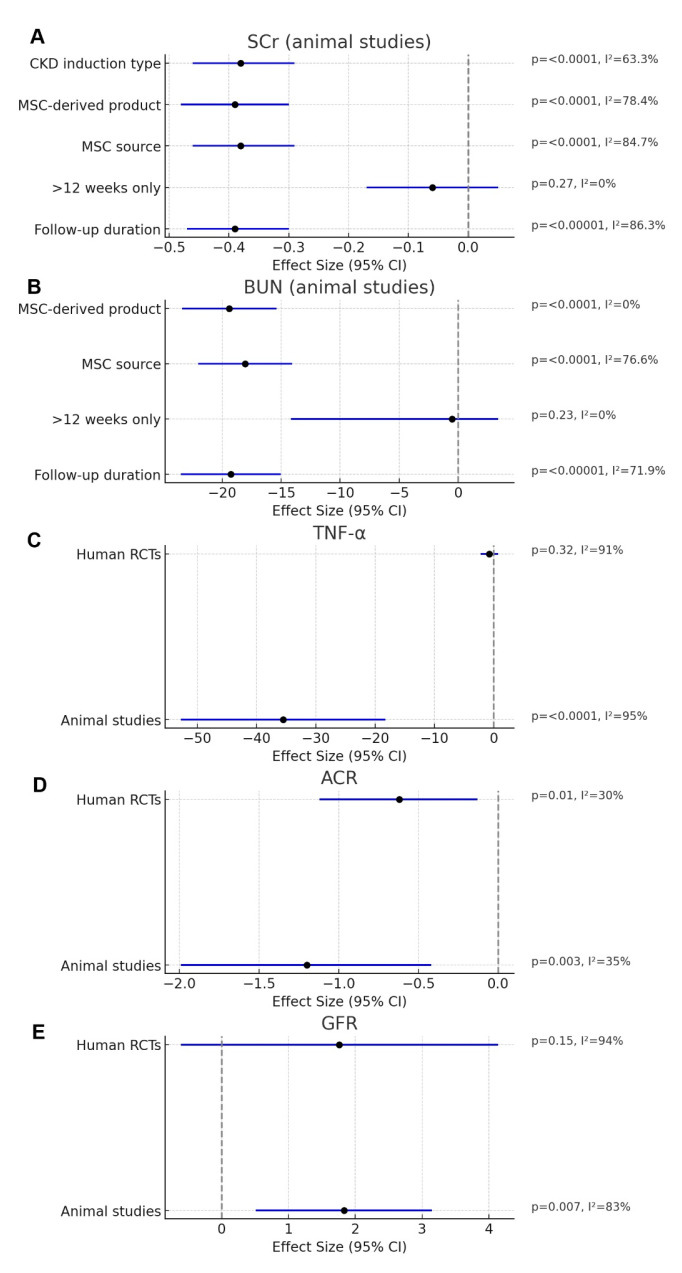
Consolidated multi-panel forest plots of MSC effects on outcomes with subgroup analyses. Multi-panel forest plots summarizing the pooled effect sizes and 95% CIs for key outcomes and their subgroup analyses from animal studies and human RCTs. (**A**) SCr in animal studies, subgrouped by follow-up duration, MSC source, MSC-derived product, and CKD induction type; (**B**) BUN in animal studies, subgrouped by follow-up duration, MSC source, and MSC-derived product; (**C**) TNF-α levels in animal studies and human RCTs; (**D**) ACR in animal studies and human RCTs; (**E**) GFR in animal studies and human RCTs. Point estimates are shown as black dots, horizontal lines indicate 95% CIs, and the vertical dashed line represents no effect. *p*-values and heterogeneity (I^2^) statistics are shown for each analysis. Forest plots were generated in Python (v3.11), using the Matplotlib library (v3.8.2).

BUN levels were reported in 33 animal studies. Subgroup analysis according to the CKD induction type showed significant reductions in the MSC-treated group (MD = −0.38; 95% CI: −0.48 to −0.29; *p* < 0.00001; I^2^ = 64.3%) (Figure 5). Analysis by follow-up duration—0–≤2 weeks, >2–≤4 weeks, >4–≤8 weeks, >8–≤12 weeks, and >12 weeks—also showed significant BUN reductions at all time points except > 12 weeks (overall MD = −19.27; 95% CI: −23.50 to −0.30; *p* < 0.00001; I^2^ = 71.9%) (Figure S5). At >12 weeks, the effect was not significant (MD = −0.50; 95% CI: −14.19 to 3.39; *p* = 0.23; I^2^ = 0%) (Figure S6). Further subgrouping by MSC source showed significant BUN reductions (MD = −18.07; 95% CI: −22.05 to −14.09; *p* < 0.0001; I^2^ = 76.6%) (Figure S7), as did subgrouping by MSC-derived product (whole cells and exosomes: MD = −19.40; 95% CI: −23.41 to −15.38; *p* < 0.0001; I^2^ = 0%) (Figure S8). The lowest heterogeneity was observed in MSC-derived products, reinforcing their role as another potential source of heterogeneity, along with the type of CKD induction. Five animal studies were combined to evaluate GFR, showing a significant increase in the MSC-treated group compared with that in the control group (SMD = 1.83; 95% CI: 0.51 to 3.15; *p* = 0.007; I^2^ = 83%) (Figure 6A); however, the increase was not significant in three human RCTs (SMD = 1.76; 95% CI: −0.61 to 4.14; *p* = 0.15; I^2^ = 94%) (Figure 6B). Subgroup analyses for the main outcomes were consolidated into a single multi-panel figure (Figure 7). TSA for GFR in human RCTs showed that the cumulative Z-curve crossed the monitoring boundary and reached the required sample size, indicating a statistically robust increase in GFR after MSC therapy (Figure S9).

### 3.4. Urinary Protein

The included studies used various methods to measure urinary protein levels, including albuminuria, proteinuria, PCR, and ACR. The MSC-treated group exhibited a significant reduction in albuminuria (SMD = −2.42; 95% CI: −4.08 to −0.76; *p* = 0.004; I^2^ = 84%) (Figure S10a). A notable decrease in proteinuria was also observed in the MSC-treated group (SMD = −2.14; 95% CI: −3.13 to −1.15; *p* < 0.0001; I^2^ = 84%) (Figure S10b). Furthermore, both urinary PCR and ACR were significantly decreased in the MSC-treated group, with overall effect sizes of SMD = −0.69 (95% CI: −1.13 to −0.25; *p* = 0.002; I^2^ = 14%) and SMD = −1.20 (95% CI: −1.99 to −0.42; *p* = 0.003; I^2^ = 35%), respectively (Figure S11a,b). Pooled data from human RCTs also indicated a significant reduction in ACR (SMD = −0.62; 95% CI: −1.12 to −0.13; *p* = 0.01; I^2^ = 30%) (Figure S11c). TSA on ACR in human RCTs also showed that the Z-curve crossed the monitoring boundary and met the required sample size, suggesting conclusive evidence for ACR improvement (Figure S12).

### 3.5. Quality Assessment

**Figure 8:**
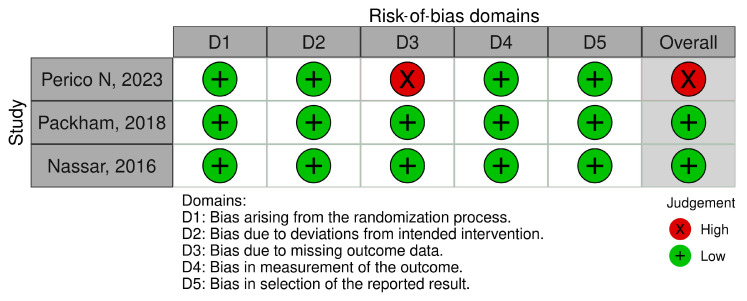
Risk-of-bias assessment of human RCTs [64,65,66] using Cochrane RoB-2. Figure generated using Review Manager (RevMan) version 5.4, the Cochrane Collaboration.

Quality assessments were conducted for the animal experiments and clinical trials (Table 3 and Figure 8). Table 3 shows the SYRCLE assessment, highlighting several “unclear” judgments regarding the quality of the animal experiments, particularly regarding allocation concealment, blinding (detection bias), and random outcome assessment. These evaluations highlight a widespread shortcoming in the implementation of randomization and blinding methods in animal studies. Figure S1 shows the RoB-2 assessment for human RCTs, with two studies having an overall low risk of bias and one showing an overall high risk of bias because of missing outcome data.

For the 49 included animal studies, SYRCLE’s tool revealed frequent methodological shortcomings. While baseline characteristics and outcome data were often adequately reported, multiple domains, particularly sequence generation, allocation concealment, random housing, and blinding of caregivers or outcome assessors, were frequently judged as unclear or high risk. More than half of the animal studies did not report any form of blinding, introducing a notable risk of both performance and detection bias. Furthermore, only a minority of studies employed random outcome assessment, increasing the possibility of measurement bias.

Among the three included RCTs, two were judged to have a low overall risk of bias, while one trial [64] was assessed as high risk due to missing outcome data, primarily related to participant attrition during the COVID-19 pandemic. As outcomes were analyzed based on available cases without imputation, this domain received a high-risk rating under the RoB-2 tool. All other domains, including randomization, intervention adherence, outcome measurement, and reporting, were considered low risk.

### 3.6. Risk of Publication Bias

Publication bias was assessed only for Scr and BUN, as these outcomes included more than 10 individual studies. For Scr, the funnel plot appeared asymmetric, and Egger’s test indicated a statistically significant intercept (*p* = 0.0003), suggesting the presence of small-study effects and potential publication bias. In contrast, for BUN, the funnel plot was visually symmetrical, and Egger’s test did not indicate significant small-study effects (*p* = 0.9424), suggesting no evidence of publication bias. The corresponding funnel plots are presented in Supplementary Figure S13.

### 3.7. Summary of Findings and Certainty of Evidence (GRADE)

A Summary of Findings table with GRADE certainty assessments was developed to evaluate the strength of the evidence across key outcomes (Table 4). Certainty ranged from high to very low. In animal studies, serum IL-6 (MD −155.8 pg/mL, 95% CI −249.1 to −62.51) and kidney tissue TGF-β mRNA expression (MD −3.63, 95% CI −5.54 to −1.72) both showed large, statistically significant reductions, but were graded as very low certainty due to high heterogeneity and small study numbers. Serum creatinine in human RCTs (MD −0.59 mg/dL, 95% CI −1.92 to 0.74) was graded as low certainty, as were GFR (SMD 1.76, 95% CI −0.61 to 4.14) and serum TNF-α (MD −0.74 pg/mL, 95% CI −2.20 to 0.73). ACR in human RCTs showed a statistically significant reduction (MD −63.59 mg/g, 95% CI −106.2 to −20.99) and was graded as high certainty. BUN in animal studies (MD −19.27 mg/dL, 95% CI −23.51 to −15.03) was graded as very low certainty, primarily due to high heterogeneity and methodological limitations in many included studies.

## 4. Discussion

Numerous preclinical and clinical studies have explored MSC therapy for CKD; however, outcomes related to inflammation, renal fibrosis, and renal function remain inconclusive. This meta-analysis identified 52 studies, including 49 animal studies and 3 RCTs, which assessed MSC efficacy in preclinical and clinical settings. This is the first study to analyze the impact of MSCs across all forms of CKD and induction methods, focusing on their anti-inflammatory properties. While MSCs show significant advantages in animal models by reducing markers, such as IL-6, TNF-α, and KIM-1, human trials remain inconsistent. For instance, Nassar et al. reported a substantial TNF-α reduction, whereas Packham et al. found no significant changes, likely because of differences in MSC type, study populations, and follow-up durations [65,66]. Limited human studies precluded analysis of IL-6 and KIM-1 levels, despite their prognostic value in CKD [67,68]. Similarly, markers, such as TGF-β could not be fully evaluated in humans, but were significantly reduced in animal studies, underscoring TGF-β’s role in kidney fibrosis [69].

Renal function, as measured by creatinine, BUN, and GFR, improved significantly in animal studies, but not in patients. This discrepancy is further supported by TSA, which revealed inconclusive findings for creatinine, but confirmed a significant effect for GFR. Urine markers, such as ACR and PCR, which are cost-effective prognostic tools for CKD, were analyzed. ACR was significantly reduced in both animal and human studies, emphasizing its utility in CKD evaluation. This finding was further corroborated by the TSA result, which confirmed a conclusive effect for ACR in human RCTs. ACR and PCR can serve as prognostic markers because of their association with several complications, including anemia, acidosis, and electrolyte imbalance [70].

MSCs improve the CKD microenvironment through various mechanisms. They modulate the inflammatory microenvironment by reducing inflammatory mediators, such as monocyte chemoattractant protein-1 and TNF-α, along with increasing anti-inflammatory factors, such as IL-10 [71,72]. Oxidative stress, which plays a crucial role in renal fibrosis, is alleviated by MSCs through improvement of the antioxidant capacity of renal cells. This leads to an improved oxidant balance in the kidney, reducing oxidative stress and potentially promoting renal function recovery [71,73]. The antifibrotic effect of MSCs, through reducing profibrotic factors such as TGF-β, also plays a major therapeutic role in CKD [74]. However, it should be noted that markers such as TGF-β and NF-κB, while commonly used in preclinical research, serve primarily as mediators of fibrosis and oxidative stress, rather than direct measurements of these pathological processes. Therefore, while changes in these markers suggest therapeutic effects, they provide an indirect quantification of fibrotic burden or oxidative damage. MSCs, which are recognized for their potential to promote tissue regeneration and repair, have also been associated with improved kidney function in patients with CKD. They develop into various cell types and release trophic factors that enhance kidney cell survival, proliferation, and angiogenesis. In CKD models, MSCs have demonstrated the capacity to improve renal function by reducing fibrosis and enabling the repair of injured renal tissue [9,73].

High statistical heterogeneity (I^2^ > 90%) observed in many pooled analyses reflects the considerable clinical and methodological diversity among included studies. This variation stems from differences in CKD induction model, animal species and strains, MSC sources, administration protocols, and follow-up durations. To address this, random-effects models were applied, and exploratory subgroup analyses were conducted. These analyses identified MSC product type, CKD induction method, and follow-up duration as key contributors to heterogeneity. MSCs and their exosomes exert renoprotective effects through distinct mechanisms: MSCs act via paracrine signaling to modulate inflammation, fibrosis, and apoptosis, whereas exosomes deliver molecular cargo that downregulates proinflammatory cytokines [75,76,77,78]. The induction method in preclinical studies significantly affects therapeutic outcomes because of variations based on model and strain. Different CKD induction methods influence disease severity and phenotypic features, which affect MSC efficacy [79,80]. Subgroup analysis by follow-up duration revealed that creatinine reductions occurred in animal studies, except those with follow-up > 12 weeks, paralleling human trials in which all follow-ups exceeded 12 weeks—suggesting a potential plateau effect. Collectively, these findings highlight the need for standardized protocols and harmonized study designs to improve reproducibility and facilitate translational application. Publication bias was observed in the creatinine data, highlighting the importance of cautious interpretation of the results. Our ongoing research project investigates the role of MSCs in repairing the CKD microenvironment and halting disease progression. The small number of human trials and heterogeneity in methodologies, including MSC administration techniques, CKD induction methods, and follow-up duration limit this analysis. Subgroup analysis reduced heterogeneity by focusing on MSC-derived products and CKD induction type. Animal studies have also shown methodological weaknesses, such as unclear randomization and blinding, which future research must address. The GRADE assessment indicated that most outcomes, particularly from animal studies, were supported by very low-certainty evidence, due to high heterogeneity and methodological limitations. Human trial data generally provided low-certainty evidence, except for ACR, which reached high certainty. These results suggest that, while MSC therapy shows potential, the current evidence, especially in humans, remains limited, and should be interpreted with caution. This review is limited by the predominance of preclinical studies relative to human RCTs, substantial methodological and clinical heterogeneity, and generally low certainty of evidence for several outcomes, which should be considered when interpreting the findings. Given the gap between animal and human findings, MSC therapy should continue to be investigated as a potential adjunct, rather than a replacement for standard CKD care, until stronger clinical evidence emerges.

## 5. Conclusions

MSCs demonstrate robust anti-inflammatory and renoprotective effects in animal models of CKD, reflected in improved biomarkers and reduced fibrosis. However, clinical evidence from randomized trials remains limited and inconsistent, with few significant effects observed on inflammatory mediators or kidney function. Future studies should prioritize larger, well-designed RCTs with standardized MSC sources and protocols; direct assessments of histological fibrosis and oxidative stress in patients; extended follow-up to assess long-term effects; and integration of advanced delivery platforms.

## Figures and Tables

**Table 1:** Characteristics of Animal Studies.

No.	Study	CKD Model	Model Features (CKD Induction)	Source of Stem Cell	Intervention Group	Comparator Group	Sample Size		Follow-Up Duration (Weeks)
							Intervention Group	Comparator Group	
1	Zhao X, et al., 2024 [16]	Adriamycin (ADR)-induced nephropathy rats	Rats were induced by intravenous injection with adriamycin (8 mg/kg)	Adipose mesenchymal stem cells (ADSCs)	ADR + ADSC group	ADR group	7	7	6
2	He J, et al., 2024 [17]	Diabetic nephropathy (DN) mice	DM was induced in male mice by intraperitoneal (i.p.) injection of 80 mg/kg STZ in 0.1 M citrate buffer, at pH 4.5, following 6 h fasting for 5 consecutive days.	Human mesenchymal stromal cells	DM mice injected i.v. with 0.2 mL containing 5 × 105 hUC-MSCs	DM mice injected i.v with 0.2 mL NS. (normal saline)	6	6	18
3	Yang C, et al., 2024 [18]	DN rats	Right nephrectomy with upper two-thirds arterial ligation of the left kidney, preserving lower-pole blood flow. DM was induced on day 7 post-CKD with STZ (30 mg/kg) and aminoguanidine (180 mg/kg), intraperitoneally.	Adipose-derived mesenchymal stem cells	Diabetic + ADMSC group	Vehicle-injected diabetic group	NA	NA	8.5
4	Ni Y, et al., 2023 [19]	DN rats	Induced by STZ (55 mg/kg)	hAMSCs were obtained from placental amniotic membranes during cesarean delivery	PBS-hAMSCs were injected slowly through the penile vein (2.0 × 106 cells/each)	PBS	6	6	12
5	Shati AA, et al., 2023 [20]	Cisplatin (CDDP)-induced nephropathy rats	Rats were injected IP with CDDP (3.2 mg per kg body weight every week for four successive weeks) to induce nephrotoxicity.	Bone marrow-derived mesenchymal stem cells (BMSCs)	CDPP + BMSCs-treated group	CDPP group	15	15	12
6	Morsy S, et al., 2022 [21]	CKD Rats	A single intravenous (IV) injection of ADR (Doxorubicin hydrochloride [50 mg/25 mL saline]) at a dose of 5 mg/kg.	Adipose-derived stem cells (ADMSCs)	IV Injection of ADMSCs (2 × 106 cells suspended in 1 mL saline) 1 week after the ADR injection	1 mL saline 1 week after adriamycin injection.	6	6	12
7	Rafiee Z, et al., 2022 [22]	DN rats	A single intraperitoneal administration of STZ (60 mg/kg).	Kidney stem cells (KSCs)	2 × 106 cells/rat of KSC IV	Normal saline	8	8	2
8	Yue Y, et al., 2022 [23]	DN rats	Induction by 5/6 nephrectomy of left kidney and right nephrectomy, followed by intraperitoneal administration of aminoguanidine (180 mg/kg) and STZ (30 mg/kg).	Human umbilical cord-derived mesenchymal stem cells (HUCDMSCs)	DKD + HUCDMSCs	DKD with no intervention	6	6	8.5
9	Alasmari WA, et al., 2022 [24]	Post-menopause CKD rats	At first, the menopause model was achieved by surgical bilateral ovariectomy in female albino rats. After that, 100 µg of exosomes was given to ovariectomized rats, and the study continued for 2 months.	Bone marrow mesenchymal stem/stromal cells (BM-MSCs)	CKD + BM-MSCs	CKD with no intervention	7	7	9
10	Miyasaki DM, 2022 [60]	Adenine-induced nephropathy	CKD was induced using adenine (0.75% in 15 g pellet chow), administered for seven consecutive days.	Human umbilical cord tissue (hUCT)-derived mesenchymal stem cells (MSCs)	hUCT-derived MSCs (MSC-EV group)	Control (saline) group	9	9	4
11	Almeida A, et al., 2022 [25]	CKD rats	Rats were put under ketamine and xylazine anesthesia (100 and 5 mg kg^−1^, respectively, I.P. injection) and underwent surgery for partial occlusion of the left renal artery with the aid of a 0.2 mm silver clip.	Bone marrow-derived mesenchymal stem cells (BMSCs)	2K1C + MSCs group	2K1C + no-intervention group	6	6	6
12	Serag WM, et al., 2022 [26]	ADR-induced nephropathy rats	Rats were i.v. injected twice with 4 mg/kg ADR on day 1 and 14.	Bone marrow mesenchymal stem cells (BMSCs)	ADR + BMSC group	ADR + no-intervention group	15	15	13
13	Alasmari WA, et al., 2022 [27]	Post-menopause CKD (PM-CKD) rats	Bilateral ovariectomy in 8-month-old female albino rats, then no treatment.	Bone marrow mesenchymal stem/stromal cells (BM-MSCs)	Post-menopausal CKD group + BM-MSCs	Post-menopausal CKD group + no intervention	8	8	8
14	Akan E, et al., 2021 [28]	Renal fibrosis rats	5/6 nephrectomy (5/6 Nx) induced.	Human amnion-derived MSC (hAMSC)	5/6 Nx + hAMSC	5/6 Nx + no-intervention group	6	6	4
15	Liu L, 2021 [11]	Renal fibrosis rats	The left abdomen was opened to locate the ureter in the lower pole of the kidney. A 4–0 suture ligated the upper pole of the ureter near the calyces, and the ureter was then removed.	Pluripotent stem cell (PSC)-derived mesenchymal stem cells (MSCs)	UUO + PSC-MSC-Exosomes (Exo-H group)	UUO group	6	6	2
16	Xia C, et al., 2021a [61]	ADR-induced nephropathy rats	The rats were injected with doxorubicin through the tail vein at a dose of 4 mg/kg body weight. In addition, the rats were injected with an identical dose again, 2 weeks later.	Bone marrow stromal cell (BMSC)	BMSC group	Adriamycin group (treated with phosphate buffer)	10	10	4
17	Xia C, et al., 2021b [62]	ADR-induced nephropathy rats	A rat AN model was induced by two injections of doxorubicin.	Bone marrow stromal cells	BMSc group	ADR group	10	10	4
18	Wang S, et al., 2021 [29]	DN rats	Fed with a high-fat high-sugar diet for one month before receiving an intraperitoneal injection of STZ.	Bone marrow mesenchymal stem cells (BMMSCs-Exos)	DN + BMMSC-Exo group	DN + no intervention group	9	9	8
19	Yea JH, et al., 2021 [30]	Adenine-induced nephropathy	Mice in the CKD groups were fed a 0.25% adenine-containing diet to induce CKD	Human adipose-derived MSCs	cExo-treated CKD (exosome without melatonin)	PBS-treated CKD group	5	5	3
20	Lin L, et al., 2020 [31]	DN rats	A single intraperitoneal injection of STZ 65 mg/kg	Bone marrow mesenchymal stem cells (BMSCs)	BMSC group	No-intervention group	10	10	6
21	Sheu JJ, et al., 2020 [32]	Nephrectomy-induced CKD rats	Animals were anesthetized with 2.0% isoflurane and placed on a warming pad for midline laparotomies. SC rats underwent laparotomy only, while CKD was induced in CKD groups by right nephrectomy and arterial ligation of the upper two-thirds of the left kidney, preserving blood flow to the lower pole, to simulate CKD.	Pluripotent stem cell (iPSC)-derived mesenchymal stem cells (MSCs)	iPS-MSC group	No-intervention group	6	6	8
22	Yu Y, et al., 2020 [33]	Aristolochic acid (AA)-induced renal fibrosis mice	Mice were intraperitoneally injected with AA at a dosage of 5 mg/kg every other day for 2 weeks.	Human umbilical cord mesenchymal stem cells (ucMSCs)	AA + MSC group	AA group	8	8	4
23	Liu B, et al., 2020 [34]	Unilateral Ureteral Obstruction (UUO)-induced renal fibrosis rats	Rats were anaesthetized with sodium pentobarbital (30 mg/kg, i.p.), in the UUO group, the left ureter was exposed and ligated with 4–0 silk thread. In the sham group, the left ureter was dissociated but not ligated.	Exosomes released by human umbilical cord mesenchymal stem cells (hucMSC-Ex)	hucMSC-Ex group (UUO treated with hucMSC-Ex (200 μg of exosomes dissolved in PBS)	UUO group	8	8	2
24	Chen L, et al., 2020 [35]	DN rats	Diabetes was induced by single intraperitoneal injection of 60 mg/kg STZ in sodium citrate buffer (0.01 M, pH 4.5) after overnight fasting; 6 weeks after STZ injection, the rats showed a blood glucose level over 16.7 mmol/L (DN).	Human umbilical cord-MSC (UC-MSC)	UC-MSCs group (DN rats injected with UC-MSCs (2 × 10^6^ cells suspended in 0.5 mL PBS)	DN group (injected with 0.5 mL PBS)	5	5	2
25	Xiang E, et al., 2020 [36]	DN rats	Diabetic rats were induced by a single intraperitoneal injection of 60 mg/kg STZ (dissolved in 0.1 M citrate buffer, pH 4.5) 4–6 weeks after STZ injection, the rats showed a blood glucose level over 16.7 mmol/L (DN)	Human umbilical cord tissue-MSC (UC-MSC)	DN + UC-MSC (2 × 10^6^/500 μL) group	DN + PBS group	5	5	6
26	An X, et al., 2019 [37]	DN rhesus macaques	Adult healthy male rhesus macaques (3–5 years) received a single high dose of STZ (80 mg/kg) intravenously, to induce diabetes, with insulin maintaining FBG at 15–20 mmol/L. To develop DN, they were fed a diet of 10 g salt and 60 g peanuts for at least 2 years.	Human umbilical cord-derived MSCs	MSC-treated (DN + MSCs) group	Normal saline-treated (DN + NS) group	6	4	52
27	Cetinkaya B, et al., 2019 [38]	CKD rats	Aristolochic acid I (AA) was used to mimic the structural and functional damage of CKD. AA was dissolved in Dimethyl sulfoxide.	Human amnion-derived mesenchymal stem cells (hAMSCs)	AA + hAMSCs group	AA group	6	6	8
28	Takemura S, et al., 2019 [39]	DN rats	SDT fatty rats, a type-2 diabetes model, were created by introducing the fa allele of Zucker rats. Right nephrectomy was performed on 5-week-old rats, under anesthesia, to accelerate DN progression.	Adipose-derived mesenchymal stem cell (ASC)	ASC iv group	Sham-operated (sham group)	7	6	2
29	Song IH, et al., 2018 [40]	ADR-induced nephropathy rats	Nephropathy was induced by ADR (4 mg/kg).	Bone marrow-derived mesenchymal stem cells (BM-derived MSCs)	ADR + MSCs (MSC group)	ADR + vehicle (CON group)	4	4	6
30	Li Y, et al., 2018 [41]	DN rats	Diabetes was induced in adult male SD rats by STZ injection (55 mg/kg, i.p.).	Bone marrow-derived mesenchymal stem cells (BM-derived MSCs)	DN + MSC group	DN + saline group	11	14	10
31	Liu B, et al., 2018 [63]	Renal fibrosis rats	SDT fatty rats, a type-2 diabetes model, were created by introducing the fa allele of Zucker rats. Unilateral nephrectomy was performed on 5-week-old rats, to accelerate DN progression.	Human umbilical cord-derived mesenchymal stem cell (hucMSC)	UUO + hucMSC group	UUO + PBS group	8	8	2
32	Rashed LA, et al., 2018 [42]	DN rats	Diabetes was induced by a single intraperitoneal injection of STZ (50 mg/kg) dissolved in freshly prepared 0.1 M citrate buffer (pH = 4.5).	Bone marrow-derived mesenchymal stem cells (MSCs)	DN + MSC group	DN + no intervention group	10	10	6
33	Pan XH, et al., 2017 [43]	UUO-induced renal fibrosis rabbits	The left kidney and ureter were isolated, and a 2 cm section of the ureter was ligated with a 5–0 suture.	Induced mesenchymal stem cells (iMSCs) from adult peripheral blood mononuclear cells (PBMCs)	UUO + induced PBMCs (iMSCs) group	UUO animals that did not undergo any transplantation	10	10	4
34	Lang H, et al., 2016 [44]	DN rats	After adaptive feeding for 1 week and fasting 12 h, the model group was given STZ55 mg/kg by i.p. injection.	Bone marrow mesenchymal stem cells (BMSCs)	DN + MSC group	DN + no intervention (culture medium) group	10	10	12
35	Jia X, et al., 2016 [45]	Adenine-induced nephropathy	Acclimated for 1 week before the experiment, CRF animals were given 2% adenine suspension every morning by gavage, at a dose of 200 mg/(kg·d).	Bone marrow mesenchymal stromal cells (BM-MSCs)	Model + BM-MSCs group	Model + PBS group	12	12	8
36	Anan HH, et al., 2016 [46]	ADR-induced nephropathy rats	Injection of a single dose of Adriamycin (15 mg/kg) intraperitoneally.	Bone marrow-derived mesenchymal stromal cells (BM-MSCs)	The adriamycin + MSC-treated group (Group IV)	Adriamycin-treated groups (Group II)	5	8	5
37	Moghadasali R, et al., 2015 [47]	Cisplatin-induced CKD monkey model	Administration of single dose of cisplatin (5 mg/kg), intravenously.	Autologous mesenchymal stromal cells (MSCs)	Cisplatin + MSCs	Cisplatin (day 0) + intrarenal arterial injection of NS, 4 days after	3	3	36
38	Caldas HC, 2015 [48]	Renal mass reduction-induced CKD rats	Renal mass reduction was performed using two models: 5/6 reduction for severe CRF and 2/3 reduction for less severe CRF.	Bone marrow cells isolated from the femur and tibiae of male Wistar rats	MSC5/6 group	CRF5/6 group	10	10	12 after surgery
39	da Silva AF, et al., 2015 [49]	UUO-induced chronic renal fibrosis rats	The UUO procedure involved anesthetizing rats, ligating the ureter or performing sham surgery, followed by administering MSCs or MSC-CM via the cava vein. Animals were monitored and euthanized at 7 or 14 days, for analysis.	Bone marrow-derived mesenchymal stromal cells (BM-MSCs)	UUO + MSC (UUO with mesenchymal stem cells)	UUO group	7	7	2 post surgery
40	Pan XH, et al., 2014 [50]	DN treeshrews	Administering an intraperitoneal injection of STZ at a dose of 100 mg/kg, after fasting.	Bone marrow-derived mesenchymal stromal cells (BM-MSCs)	DN + BM-MSC group	DN group	6	6	12
41	Abdel Aziz MT, et al., 2014 [51]	DN rats	A single intraperitoneal injection of STZ (60 mg/kg) was dissolved in 0.1 mol/L citrate buffer (pH 4.5).	Bone marrow-derived mesenchymal stromal cells (BM-MSCs)	DN + BM-MSC rats	DN + IV PBS	20	20	4
42	LV Sha-sha, et al., 2013 [52]	DN rats	Single intra-peritoneal injection of STZ (60 mg/kg) after one night’s fasting.	Bone marrow-derived mesenchymal stromal cells (BM-MSCs)	DN + MSC group	DN group (IV 0,9% Saline)	16	16	8
43	Ma H, et al., 2013 [53]	ADR-induced nephropathy rats	Administering adriamycin hydrochloride at a dosage of 4 mg/kg on day 1 and 3.5 mg/kg on day 8, via the tail vein.	Human umbilical cord mesenchymal stem cells (HuMSCs)	ADR + MSC IV	ADR group	6	6	12
44	Villanueva S, et al., 2011 [54]	Nephrectomy-induced CKD rats	Rats underwent nephrectomy under ketamine/xylazine anesthesia, with kidney mass reduced by clamping two renal artery subdivisions, and contralateral nephrectomy one week later to induce kidney damage over 5 weeks.	Bone marrow-derived mesenchymal stromal cells (BM-MSCs)	NPX + MSC group	NPX group	7	7	5
45	Jiao YQ, et al., 2011 [55]	ADR-induced nephropathy rats	Injected with 0.25 mg ADR/100 g body weight in 2 mL of saline on days 1 and 21, followed by an injection with 2 mL of PBS eight weeks after the second dose of ADR.	Metanephric mesenchymal cells (MMCs)	ADR glomerulopathy + MMC group	ADR glomerulopathy group	15	15	16
46	Zhou H, et al., 2009 [56]	DN rats	Received a single intraperitoneal injection of STZ (60 mg/kg), dissolved immediately before administration in freshly prepared 0.1 mol/L citrate buffer (pH 4.5).	Bone marrow-derived mesenchymal stromal cells (BM-MSCs)	MSC Group (MSC-treated group)	CsA (Cyclosporin A)-treated group	16	16	8
47	Choi S, et al., 2009 [57]	Nephrectomy-induced CKD	Rats underwent a modified 5/6 nephrectomy: full right nephrectomy and left renal artery/vein clamping for 40 min, followed by half nephrectomy.	Bone marrow- derived mesenchymal stem cells (MSCs)	CRF + MSC Group	CRF group	6	3	24
48	Caldas HC, et al., 2008 [58]	Renal mass reduction-induced CKD rats	Rats underwent 5/6 renal mass reduction.	Bone marrow mesenchymal cells (MSCs) isolated from the femoral and tibial bones.	MSC group	Control group, underwent intrarenal parenchymal injections of 0.15 mL medium	5	5	16
49	Ninichuk, et al., 2006 [59]	Collagen4A3-deficient mice model for CKD (COL4A3)	COL4A3-deficient mice (129/SvJ background) were bred under pathogen-free conditions, with genotypes confirmed by PCR. These mice develop glomerulosclerosis, renal fibrosis, and uremia-related death by ~10 weeks of age.	Bone marrow-derived mesenchymal stem cells (MSCs)	COL4A3 + MSC Group	COL4A3 + Saline Group	10	10	9.3

ADMSC, Adipose-Derived Mesenchymal Stem Cells; ADR, Adriamycin; ASC, Adipose-Derived Stem Cell; BM-MSCs, Bone Marrow-Derived Mesenchymal Stem Cells; BMMSC-Exos, Bone Marrow Mesenchymal Stem Cell-Derived Exosomes; CKD, Chronic Kidney Disease; CRF, Chronic Renal Failure; DM, Diabetes Mellitus; HUCDMSCs: Human Umbilical Cord-Derived Mesenchymal Stem Cells; hAMSCs, Human Amnion-Derived Mesenchymal Stem Cells; hUC-MSC, Human Umbilical Cord Mesenchymal Stem Cells; iPSC, Induced Pluripotent Stem Cells; IV, Intravenous; MSC, Mesenchymal Stem Cell; MSC-CM; Mesenchymal Stem Cell-Conditioned Media; NPX, Nephrectomy: NS, Normal Saline; PBS, Phosphate-Buffered Saline; STZ, Streptozotocin; UUO, Unilateral Ureteral Obstruction.

**Table 2:** Characteristics of Human RCTs.

No.	Study	Study Design	Type of Patients	Study Subjects	Source of Stem Cell	Intervention Group	Comparator Group	Sample Size		Follow-Up Duration (Weeks)
								Intervention Group	Comparator Group	
1	Perico N, et al., 2023 [64]	RCT	Diabetic nephropathy (DN) patients	Participants must be 40–85 years old, with type 2 diabetes for at least three years. They must have a UACR of ≥88 mg/g (≥10 mg/mmol) and an eGFR of 25–55 mL/min/1.73 m^2^ (CKD-EPI equation), confirmed by at least two measurements 30+ days apart in the past 6 months. They should have a documented eGFR decline of ≥10 mL/min/1.73 m^2^ over 3 years, ≥5 mL/min/1.73 m^2^ per year (three readings 90+ days apart in 18 months), or an intermediate/high 5-year risk of kidney failure (Tangri equation, CKD stage 3–5).	ORBCEL-M (healthy donor bone-marrow aspirates)	80 × 10^6^ cells ORBCEL-M of administered IV into a peripheral arm vein	Cryostor CS10	12	4	72
2	Packham DK, et al., 2018 [65]	RCT	DN patients	Male and female patients aged 45–85 years with type 2 diabetes and advanced diabetic nephropathy (eGFR 20–50 mL/min/1.73 m^2^) on a stable standard regimen of the maximum tolerated dose of an ACE inhibitor or ARB for at least three months before screening.	Bone marrow-derived mononuclear cells from healthy paid adult donors (U.S. adopted name rexlemestrocel-L)	150 × 10^6^ Rexlemestrocel-L IV	Saline	10	20	12
3	Nassar W, et al., 2016 [66]	RCT	CKD	Participants must be 26–44 years old with a chronic kidney disease (CKD) diagnosis for over six months, an eGFR of 15–60 mL/min, normal liver function, and no chronic or recurrent infections in the past 12 months.	Human cord blood mesenchymal stem cells (hCB-MSCs)	Two doses of MSC-EVs, intra-arterial and intravenous injections at 100 μg/kg/dose	Saline	20	20	48

Cryostor CS10, Cryopreservation Solution 10%; CKD-EPI, Chronic Kidney Disease Epidemiology Collaboration Equation; eGFR, Estimated Glomerular Filtration Rate; hCB-MSCs, Human Cord Blood Mesenchymal Stem Cells; MSC-EVs, Mesenchymal Stem Cell-Derived Extracellular Vesicles; ORBCEL-M, Specific Mesenchymal Stem Cell Product from Bone Marrow; RCT, Randomized Controlled Trial; UACR, Urine Albumin-to-Creatinine Ratio.

**Table 3:** Risk-of-bias assessment of animal intervention studies using SYRCLE.

No.	Study	Domains									
		Sequence Generation	Baseline Characteristics	Allocation Concealment	Random Housing	Blinding (Performance Bias)	Blinding (Detection Bias)	Random Outcome Assessment	Incomplete Outcome Data	Selective Outcome Reporting	Other Sources of Bias
1	Zhao X, et al., 2024 [16]	Unclear	Low	Unclear	Unclear	Unclear	Unclear	Unclear	Unclear	Low	Low
2	He J, et al., 2024 [17]	Unclear	Low	Low	Low	Low	Low	Low	Unclear	Low	Low
3	Yang C, et al., 2024 [18]	High	Low	Low	High	Unclear	Low	Low	Low	Unclear	Low
4	Ni Y, et al., 2023 [19]	Low	Low	Low	Unclear	Low	Unclear	Low	Low	Low	Low
5	Shati AA, et al., 2023 [20]	Low	Low	Unclear	Unclear	Unclear	Unclear	Unclear	Unclear	Low	Low
6	Morsy S, et al., 2022 [21]	Low	Low	Unclear	Unclear	Unclear	Unclear	Low	Low	Low	Low
7	Rafiee Z, et al., 2022 [22]	Low	Low	Unclear	Unclear	Unclear	Unclear	Low	Low	Low	Low
8	Yue Y, et al., 2022 [23]	Low	High	Unclear	Unclear	Low	Low	Unclear	Unclear	Unclear	Unclear
9	Alasmari WA, et al., 2022 [24]	Low	Low	Unclear	Low	Unclear	Unclear	Unclear	Unclear	Low	Unclear
10	Miyasaki DM, 2022 [60]	Low	Unclear	Unclear	Unclear	High	Unclear	Unclear	Low	Low	Low
11	Almeida A, et al., 2022 [25]	Unclear	Unclear	Unclear	Low	High	Unclear	Unclear	Low	Low	Low
12	Serag WM, et al., 2022 [26]	Unclear	Low	Unclear	Low	High	Unclear	Unclear	Unclear	Low	High
13	Alasmari WA, et al., 2022 [27]	Unclear	Low	Unclear	Low	High	Unclear	Unclear	Unclear	Low	High
14	Akan E, et al., 2021 [28]	High	Unclear	Unclear	Low	High	Unclear	Unclear	Low	Low	Low
15	Liu L, 2021 [11]	Unclear	Low	Unclear	High	Unclear	High	High	Unclear	Unclear	Unclear
16	Xia C, et al., 2021a [61]	Low	Low	Unclear	Unclear	Low	Low	Low	Low	Low	High
17	Xia C, et al., 2021b [62]	Low	Low	Unclear	High	Unclear	Unclear	High	Unclear	Unclear	Unclear
18	Wang S, et al., 2021 [29]	Low	Low	Unclear	Unclear	Unclear	Unclear	Unclear	Low	Low	High
19	Yea JH, et al., 2021 [30]	Low	High	Unclear	Unclear	Unclear	Unclear	Unclear	Low	Low	High
20	Lin L, et al., 2020 [31]	Unclear	High	Unclear	Low	Unclear	Unclear	Unclear	Low	Low	Unclear
21	Sheu JJ, et al., 2020 [32]	Unclear	Low	Unclear	Unclear	Unclear	Unclear	Unclear	Unclear	Low	Unclear
22	Yu Y, et al., 2020 [33]	Low	Unclear	Low	Low	Unclear	Unclear	Unclear	Unclear	Low	High
23	Liu B, et al., 2020 [34]	Unclear	Unclear	Unclear	Unclear	Unclear	Unclear	Unclear	Unclear	Low	High
24	Chen L, et al., 2020 [35]	Unclear	Unclear	Low	High	High	High	Unclear	Unclear	Low	High
25	Xiang E, et al., 2020 [36]	Unclear	Low	Low	Unclear	Low	Low	Low	Unclear	Low	High
26	An X, et al., 2019 [37]	Unclear	Low	Unclear	Unclear	Unclear	Unclear	Low	Low	Unclear	Unclear
27	Cetinkaya B, et al., 2019 [38]	Low	Low	Unclear	Unclear	High	High	Unclear	High	High	High
28	Takemura S, et al., 2019 [39]	High	Unclear	Low	Low	High	Low	Unclear	High	High	Low
29	Song IH, et al., 2018 [40]	Low	Low	Unclear	Low	Unclear	High	Unclear	Unclear	Unclear	High
30	Li Y, et al., 2018 [41]	Unclear	High	Unclear	Unclear	High	Unclear	Unclear	High	Low	High
31	Liu B, et al., 2018 [63]	Unclear	Unclear	Unclear	Unclear	High	Unclear	Unclear	Unclear	Low	High
32	Rashed LA, et al., 2018 [42]	Unclear	Low	Unclear	High	Unclear	Unclear	Unclear	Unclear	Low	High
33	Pan XH, et al., 2017 [43]	Unclear	Unclear	Unclear	High	Unclear	Unclear	Unclear	Unclear	Low	High
34	Lang H, et al., 2016 [44]	Unclear	Low	High	Unclear	High	Unclear	Unclear	Unclear	Unclear	High
35	Jia X, et al., 2016 [45]	Low	Unclear	High	Unclear	High	Unclear	Unclear	Unclear	Unclear	High
36	Anan HH, et al., 2016 [46]	Unclear	Low	High	Unclear	High	Unclear	Unclear	High	Unclear	Unclear
37	Moghadasali R, et al., 2015 [47]	Unclear	Unclear	Unclear	Unclear	Unclear	Low	Low	Unclear	Unclear	Unclear
38	Caldas HC, 2015 [48]	Unclear	Unclear	High	Unclear	Low	Unclear	Unclear	High	Unclear	High
39	da Silva AF, et al., 2015 [49]	Low	Low	Unclear	Low	Unclear	Unclear	Unclear	Low	Unclear	High
40	Pan XH, et al., 2014 [50]	Unclear	Low	Unclear	Unclear	Unclear	Unclear	Unclear	Low	Unclear	High
41	Abdel Aziz MT, et al., 2014 [51]	Unclear	Unclear	Unclear	Unclear	High	High	High	Low	Low	Unclear
42	LV Sha-sha, et al., 2013 [52]	Unclear	Low	Unclear	Unclear	Unclear	Low	Low	Unclear	Unclear	Unclear
43	Ma H, et al., 2013 [53]	Unclear	Unclear	High	Unclear	High	Low	Unclear	High	Unclear	High
44	Villanueva S, et al., 2011 [54]	Unclear	Unclear	Unclear	Unclear	Low	Unclear	Unclear	Low	Low	High
45	Jiao YQ, et al., 2011 [55]	Unclear	Low	Unclear	Unclear	Unclear	Low	Unclear	Low	High	Unclear
46	Zhou H, et al., 2009 [56]	Unclear	Low	Unclear	Unclear	Low	Unclear	Unclear	Low	Unclear	High
47	Choi S, et al., 2009 [57]	High	High	High	High	High	High	Unclear	High	Unclear	High
48	Caldas HC, et al., 2008 [58]	Low	Low	Low	Unclear	Unclear	Unclear	Unclear	Low	Unclear	Unclear
49	Ninichuk, et al., 2006 [59]	Unclear	Unclear	Unclear	Low	Unclear	Unclear	Unclear	Low	Unclear	Unclear

**Table 4:** Summary of findings with GRADE certainty assessment.

Certainty Assessment								№ of Patients		Effect Size (95% CI)	Certainty
Outcome	№ of Studies	Study Design	Risk of Bias	Inconsistency	Indirectness	Imprecision	Other Considerations	MSC	Control		
Serum IL-6 in Animal Study (pg/mL)	7	non-randomized studies	not serious	very serious ^a^	not serious	very serious ^b^	strong association	54	54	MD 155.8 lower (249.1 lower to 62.51 lower)	⨁◯◯◯ Very low ^a,b^
Kidney Tissue TGF-β in Animal Study (mRNA expression)	7	non-randomized studies	not serious	very serious ^a^	not serious	not serious	strong association	49	50	MD 3.63 lower (5.54 lower to 1.72 lower)	⨁◯◯◯ Very low ^a^
Serum Creatinine in Human Study (mg/dL)	3	randomized trials	not serious	very serious ^a^	not serious	not serious	none	42	44	MD 0.59 lower (1.92 lower to 0.74 higher)	⨁⨁◯◯ Low ^a^
BUN in Animal Studies (mg/dL)	3	non-randomized studies	serious	very serious ^a^	not serious	not serious	publication bias strongly suspected, strong association ^c^	259	263	MD 19.27 lower (23.51 lower to 15.03 lower)	⨁◯◯◯ Very low ^a,c^
GFR in Humans RCT	3	randomized trials	not serious	very serious ^a^	not serious	not serious	none	42	44	SMD 1.76 higher (0.61 lower to 4.14 higher)	⨁⨁◯◯ Low ^a^
Serum TNF-α in Humans (pg/mL)	2	randomized trials	not serious	very serious ^a^	not serious	not serious	none	30	40	MD 0.74 lower (2.2 lower to 0.73 higher)	⨁⨁◯◯ Low ^a^
ACR in Humans RCT	2	randomized trials	not serious	not serious	not serious	not serious	strong association	30	40	MD 63.59 lower (106.2 lower to 20.99 lower)	⨁⨁⨁⨁ High

CI: confidence interval; MD: mean difference; SMD: standardized mean difference. Explanations: ^a^ High heterogeneity. ^b^ Wide confidence intervals. ^c^ Many key methodological safeguards—such as sequence generation, allocation concealment, random housing, and blinding of caregivers or outcome assessors—were often unclear or at high risk, raising the likelihood of bias. The absence of blinding in over half of the animal studies and limited use of random outcome assessment further increase the potential for performance, detection, and measurement bias.

## Data Availability

The data used in this meta-analysis were extracted from published studies. Individual patient-level data are not available; for access to such data, please contact the authors of the original studies directly.

## Supplementary Materials

The following supporting information can be downloaded at: https://www.mdpi.com/article/10.3390/cells14171313/s1, Figure S1: TSA Analysis for TNF-α in human RCTs; Figure S2: Forest plot of the effects of MSC treatment on serum creatinine (mg/dL) in animal studies with subgroup analysis based on duration of follow-up (0 to ≤2 weeks; >2 to ≤4 weeks; >4 to ≤8 weeks; >8 to ≤12 weeks; and >12 weeks); Figure S3: TSA Analysis for serum creatinine; Figure S4: Forest plot of the effects of MSC treatment on serum creatinine (mg/dL) in animal studies with subgroup analysis based on source of MSC (umbilical cord, bone marrow, amnion, adipose tissue, and others); Figure S5: Forest plot of the effects of MSC treatment on serum creatinine (mg/dL) in animal studies with subgroup analysis based on MSC-derived product (whole cell and exosome); Figure S6: Forest plot of the effect of MSC treatment on BUN (mg/dL) in animal studies with subgroup analysis based on duration of follow-up (0 to ≤2 weeks; >2 to ≤4 weeks; >4 to ≤8 weeks; >8 to ≤12 weeks; and >12 weeks); Figure S7: Forest plot of the effect of MSC treatment on BUN (mg/dL) in animal studies with subgroup analysis based on source of MSC (umbilical cord, bone marrow, amnion, adipose tissue, and others); Figure S8: Forest plot of the effect of MSC treatment on BUN (mg/dL) in animal studies with subgroup analysis based on MSC-derived product (whole cell and exosome); Figure S9: TSA Analysis for GFR in human RCTs; Figure S10: Forest plots of the effect of MSC treatment on (a) Albuminuria (mg/24 h) in animal studies, (b) Proteinuria (mg/24 h) in animal studies; Figure S11: Forest plots of the effect of MSC treatment on (a) PCR in animal studies, (b) ACR in animal studies, (c) ACR in human RCTs; Figure S12: TSA Analysis for ACR in human RCTs; Figure S13: Publication bias assessment using funnel plots of (a) Scr outcome, showing asymmetry, with Egger’s test indicating significant small-study effects (*p* = 0.0003), suggesting potential publication bias; (b) BUN outcome, appearing symmetrical, with Egger’s test showing no significant small-study effects (*p* = 0.9424), suggesting no evidence of publication bias; Table S1: Search strategy; Table S2: Studies excluded after full-text screening, with corresponding reasons for exclusion.
