# Supplementary material for: Mesenchymal Stem Cells as Anti-Inflammatory Agents in Chronic Kidney Disease: A Systematic Review and Meta-Analysis
Source: Cells. 2025 Aug 24;14(17):1313. doi: 10.3390/cells14171313 (PMC12428256; doi:10.3390/cells14171313)
Supplement: Supplementary file 1 [file cells-14-01313-s001.zip › cells-3775108-supplementary.pdf]

Supplementary table

Table S1. Search strategy

1. **PubMed** (Searches done on 07/09/2024)

No.	Query	Results
1.	("renal insufficiency"[MeSH Terms] OR "renal insufficiency, chronic"[MeSH Terms] OR "kidney failure, chronic"[MeSH Terms] OR "renal dialysis"[MeSH Terms] OR "kidney transplantation"[MeSH Terms]) OR "chronic kidney disease"[Title/Abstract] OR "CKD"[Title/Abstract] OR "chronic renal disease"[Title/Abstract] OR "chronic renal failure"[Title/Abstract] OR "chronic kidney failure"[Title/Abstract] OR "kidney insufficiency"[Title/Abstract] OR "renal insufficiency"[Title/Abstract] OR "nephropathy"[Title/Abstract] OR "chronic renal insufficiency"[Title/Abstract] OR "kidney transplant"[Title/Abstract] OR "renal transplant"[Title/Abstract] OR "dialysis"[Title/Abstract] OR "hemodialysis"[Title/Abstract] OR "peritoneal dialysis"[Title/Abstract]	522,898
2.	("stem cells"[MeSH Terms] OR "stem cell research"[MeSH Terms] OR "mesenchymal stem cells"[MeSH Terms] OR "stem cell transplantation"[MeSH Terms]) OR ("mesenchymal stem cells"[Title/Abstract] OR "MSC"[Title/Abstract] OR "bone marrow derived msc"[Title/Abstract] OR "adipose derived msc"[Title/Abstract] OR "cellular therapy"[Title/Abstract] OR "human umbilical cord msc"[Title/Abstract])	370,280
3.	("inflammation"[MeSH Terms] OR "interleukin 6"[MeSH Terms] OR "tumor necrosis factor alpha"[MeSH Terms] OR "Fibrosis"[MeSH Terms] OR "peritoneal fibrosis"[MeSH Terms] OR "transforming growth factor beta"[MeSH Terms] OR "kidney function tests"[MeSH Terms] OR "Creatinine"[MeSH Terms] OR "blood urea nitrogen"[MeSH Terms] OR "glomerular filtration rate"[MeSH Terms] OR "proteinuria"[MeSH Terms] OR "albuminuria"[MeSH Terms] OR "safety"[MeSH Terms] OR "drug-related side effects and adverse reactions"[MeSH Terms]) OR ("inflammation"[Title/Abstract] OR "anti inflammation"[Title/Abstract] OR "IL-6"[Title/Abstract] OR "tnf alpha"[Title/Abstract] OR "renal fibrosis"[Title/Abstract] OR "tfg beta"[Title/Abstract] OR "renal function"[Title/Abstract] OR "Creatinine"[Title/Abstract] OR "blood urea nitrogen"[Title/Abstract] OR "BUN"[Title/Abstract] OR "glomerular filtration rate"[Title/Abstract] OR "eGFR"[Title/Abstract] OR "albumin creatinine ratio"[Title/Abstract] OR "ACR"[Title/Abstract] OR "protein albumin ratio"[Title/Abstract] OR "PCR"[Title/Abstract] OR "safety"[Title/Abstract] OR "adverse event"[Title/Abstract])	3,237,412
4.	#1 AND #2 AND #3	1,501
5.	#4, Filter language: English	1,446

2. **Cochrane** (Searches done on 08/08/2025) Filter: time limited to 07/09/2024 and English Language only)

No.	Query	Results
1.	MeSH descriptor: [Renal Insufficiency] explode all trees	13584
2.	MeSH descriptor: [Renal Insufficiency, Chronic] explode all trees	10021
3.	MeSH descriptor: [Kidney Failure, Chronic] explode all trees	6170
4.	MeSH descriptor: [Renal Dialysis] explode all trees	7457
5.	MeSH descriptor: [Kidney Transplantation] explode all trees	4674
6.	(chronic kidney disease):ti,ab,kw OR (CKD):ti,ab,kw OR (chronic renal disease):ti,ab,kw OR (chronic renal failure):ti,ab,kw OR (chronic kidney failure):ti,ab,kw	27519
7.	(kidney insufficiency):ti,ab,kw OR (renal insufficiency):ti,ab,kw OR (nephropathy):ti,ab,kw OR (chronic renal insufficiency):ti,ab,kw OR (kidney transplant):ti,ab,kw	24613
8.	(renal transplant):ti,ab,kw OR (dialysis):ti,ab,kw OR (hemodialysis):ti,ab,kw OR (peritoneal dialysis):ti,ab,kw	30599
9.	#1 OR #2 OR #3 OR #4 OR #5 OR #6 OR #7 OR #8	60575
10.	MeSH descriptor: [Stem Cells] explode all trees	1334
11.	MeSH descriptor: [Stem Cell Research] explode all trees	0
12.	MeSH descriptor: [Mesenchymal Stem Cells] explode all trees	372
13.	MeSH descriptor: [Stem Cell Transplantation] explode all trees	3546
14.	(mesenchymal stem cells):ti,ab,kw OR (MSC):ti,ab,kw OR (bone marrow derived msc):ti,ab,kw OR (adipose derived msc):ti,ab,kw OR (cellular therapy):ti,ab,kw	9337
15.	(human umbilical cord msc):ti,ab,kw	171
16.	#10 OR #11 OR #12 OR #13 OR #14 OR #15	13075
17.	#9 AND #16	659
18.	MeSH descriptor: [Inflammation] explode all trees	16654
19.	MeSH descriptor: [Interleukin-6] explode all trees	4411
20.	MeSH descriptor: [Tumor Necrosis Factor-alpha] explode all trees	4324
21.	MeSH descriptor: [Fibrosis] explode all trees	8530
22.	MeSH descriptor: [Peritoneal Fibrosis] explode all trees	6

23.	MeSH descriptor: [Transforming Growth Factor beta] explode all trees	616
24.	MeSH descriptor: [Kidney Function Tests] explode all trees	5689
25.	MeSH descriptor: [Creatinine] explode all trees	4832
26.	MeSH descriptor: [Blood Urea Nitrogen] explode all trees	634
27.	MeSH descriptor: [Glomerular Filtration Rate] explode all trees	3969
28.	MeSH descriptor: [Proteinuria] explode all trees	2953
29.	MeSH descriptor: [Albuminuria] explode all trees	1700
30.	MeSH descriptor: [Safety] explode all trees	5012
31.	MeSH descriptor: [Drug-Related Side Effects and Adverse Reactions] explode all trees	5151
32.	(inflammation):ti,ab,kw OR (anti inflammation):ti,ab,kw OR (IL-6):ti,ab,kw OR (tnf alpha):ti,ab,kw OR (renal fibrosis):ti,ab,kw	73544
33.	(tgf beta):ti,ab,kw OR (renal function):ti,ab,kw OR (Creatinine):ti,ab,kw OR (blood urea nitrogen):ti,ab,kw OR (BUN):ti,ab,kw	55339
34.	(glomerular filtration rate):ti,ab,kw OR (eGFR):ti,ab,kw OR (albumin creatinine ratio):ti,ab,kw OR (ACR):ti,ab,kw OR (protein albumin ratio):ti,ab,kw	30726
35.	(PCR):ti,ab,kw OR (safety):ti,ab,kw OR (adverse event):ti,ab,kw	366300
36.	#18 OR #19 OR #20 OR #21 OR #22 OR #23 OR #24 OR #25 OR #26 OR #27 OR #28 OR #29 OR #30 OR #31 OR #32 OR #33 OR #34 OR #35	486190
37.	#17 AND #36	496

3. **Scopus** (Searches done on 07/09/2024)

No.	Query	Results
1.	TITLE-ABS-KEY ("chronic kidney disease" OR CKD OR "chronic renal disease" OR "chronic renal failure" OR "chronic kidney failure" OR "kidney insufficiency" OR "renal insufficiency" OR "nephropathy" OR "chronic renal insufficiency" OR "kidney transplant" OR "renal transplant" OR "dialysis" OR "hemodialysis" OR "peritoneal dialysis")	609,539
2.	TITLE-ABS-KEY ("Mesenchymal Stem Cell*" OR MSC OR "Bone Marrow-Derived MSC" OR "Adipose-Derived MSC" OR "Cellular Therapy" OR "Human umbilical cord MSC")	136,943
3.	TITLE-ABS-KEY (inflammation OR "anti inflammation" OR "IL-6" OR "TNF Alpha" OR "Renal Fibrosis" OR fibrosis OR "TGF beta" OR	5,208,424

	"Renal Function" OR creatinine OR "Blood Urea Nitrogen" OR bun OR "Glomerular filtration rate" OR egfr OR "Albumin Creatinine Ratio" OR acr OR "Protein Albumin Ratio" OR pcr OR safety OR "adverse event*")	
4.	#1 AND #2 AND #3	940
5.	#4, Filter document type: Article, Filter language: English	526

4. **ScienceDirect** (Searches done on 07/09/2024)

No.	Query	Results
1.	("chronic kidney disease" OR CKD OR "chronic renal disease" OR "renal failure")	54,158
2.	("Mesenchymal Stem Cell" OR MSC OR "Bone Marrow-Derived MSC" OR "Adipose-Derived MSC" OR "Human umbilical cord MSC")	27,508
3.	#1 AND #2	126
4.	#3, Filter article type: Research articles	67

5. **Google Scholar** (Searches done on 10/09/2024)

No.	Query	Results
1.	allintitle: ("chronic kidney disease" OR CKD OR "chronic renal disease" OR "renal failure")	55,200
2.	Allintitle: ("Mesenchymal Stem Cell*" OR MSC OR "Bone Marrow-Derived MSC" OR "Adipose-Derived MSC" OR "Human umbilical cord MSC")	47,100
3.	#1 AND #2	20

Table S2. Studies excluded after full-text screening with corresponding reasons for exclusion

No.	Author, Year	Title	Reason for Exclusion
1	Elhuisseiny, 2024	BONE MARROW-DERIVED MESENCHYMAL STEM CELL THERAPY IN CHRONIC KIDNEY DISEASE: AN EARLY PHASE CLINICAL TRIAL	Different intervention: compared two dosing regimens with different administration durations rather than one intervention versus placebo or standard care.
2	Zhao JL, 2024	BMSC-derived Exosomes Ameliorate Peritoneal Dialysis-associated Peritoneal Fibrosis via the Mir-27a-3p/TP53 Pathway.	Different outcome. The assessed outcome was related to peritoneal function and structure, not kidney-related parameters.
3	Liu, 2023	A randomized, double-blind controlled clinical study of human umbilical cord mesenchymal stem cells (UC-MSC) delaying renal function progression in patients with chronic kidney disease (CKD3, Stage 4)	Study protocol with no results issued yet.
4	Griffin, 2022	Safety and Preliminary Efficacy Results of a Novel Mesenchymal Stromal Cell Therapy in Diabetic Kidney Disease: the Multicenter, Randomized, Placebo-Controlled, Phase-1b/2a NEPHSTROM Clinical Trial	Overlapping data with a study by Perico, 2023.
5	Griffin, 2022	Mesenchymal Stem/Stromal Cells: INTERIM REPORT FROM THE NEPHSTROM MULTI-CENTRE, RANDOMISED, DOUBLE-BLIND, PLACEBO-CONTROLLED PHASE-1B CLINICAL TRIAL OF A NOVEL MESENCHYMAL STROMAL CELL THERAPY IN PROGRESSIVE DIABETIC KIDNEY DISEASE	Overlapping data with a study by Perico, 2023.
6	Stavas J, 2022	Novel Renal Autologous Cell Therapy for Type 2 Diabetes Mellitus Chronic Diabetic Kidney Disease: clinical Trial Design	Study protocol with no results issued yet.
7	Zeng, 2022	Human Umbilical Cord Mesenchymal Stem Cell(UC-MSC)Delayed Renal Chronic Kidney Disease (CKD 3-4)	Study protocol with no results issued yet.
8	Xiaozhou, 2022	The clinical study of mesenchymal stem cells in the treatment of early diabetic nephropathy	Study protocol with no results issued yet.

9	Yu F, 2023	Bone Marrow Mesenchymal Stem Cell-Derived Exosomes Alleviate Peritoneal Dialysis-Associated Peritoneal Injury.	Different outcome. The assessed outcome was peritoneal structural and functional changes: not kidney-related parameters.
10	Sedeak, 2021	Role of stem cell therapy in diabetic nephropathy in rats: Biochemical, histological and immunohistochemical study	Unit of measurement was not specified.
11	Kheradmand, 2020	Protective Effect of Wharton's Jelly-derived Mesenchymal Stem Cells on Renal Fibrosis in Rats with Unilateral Ureteral Obstruction	Only one outcome was comparable, but it was reported in a different unit and measured using a different method (Delta CT by qPCR).
12	Cai-Mei Z, 2020	Role of adipose tissue-derived mesenchymal stem cells in CKD: a phase 1 study assessing safety and clinical feasibility	Single-arm study without a comparator group.
13	Costalanga EC, 2020	Adipose-Derived Mesenchymal Stem Cells Modulate Fibrosis and Inflammation in the Peritoneal Fibrosis Model Developed in Uremic Rats.	1) Different population: Peritoneal fibrosis. 2) Different outcome: samples were taken from the peritoneal membrane, not kidney tissue.
14	Wang, 2020	Clinical Research of UC-MSCs in the Treatment of Diabetic Nephropathy	Study protocol with no results issued yet.
15	Alatab, 2019	Systemic Infusion of Autologous Adipose Tissue-Derived Mesenchymal Stem Cells in Peritoneal Dialysis Patients: Feasibility and Safety.	1) No comparator group. 2) Outcomes were peritoneal samples; the study used clinical, biochemical, and peritoneal equilibration test (PET) data to evaluate solute transport in the peritoneum.
16	Unknown, 2019 (NCT04216849)	Clinical Study of Umbilical Cord Mesenchymal Stem Cells in the Treatment of Type 2 Diabetic Nephropathy	Study protocol with no results issued yet.
17	Makhlough, 2018	Bone marrow–mesenchymal stromal cell infusion in patients with chronic kidney disease: A safety study with 18 months of follow-up	Single-arm study without a comparator group.
18	Sherkacian S, 2018	Phase I clinical trial of administration of adipose-derived mesenchymal stem cells in peritoneal dialysis patients	Different outcome: Samples were taken from peritoneum for peritoneal solute transport parameters measured by Peritoneal Equilibration Test (PET).

19	Sun, 2018	Human Umbilical Cord Mesenchymal Stem Cells Treatment for Lupus Nephritis (LN)	Study protocol with no results issued yet.
20	Xu, 2018	Clinical study on the Treatment of Placenta-derived Mesenchymal Stem Cells for Diabetic Nephropathy	Study protocol with no results issued yet.
21	Sherkacian S, 2017	Phase I clinical trial of bone marrow mesenchymal stromal cells in CKD patients: 18 months follow up	Single-arm study without a comparator group.
22	Quimby, 2016	Assessment of intravenous adipose-derived allogeneic mesenchymal stem cells for the treatment of feline chronic kidney disease: a randomized, placebo-controlled clinical trial in eight cats.	Unclear data reporting
23	Burgos-Silva, 2015	Adipose Tissue-Derived Stem Cells Reduce Acute and Chronic Kidney Damage in Mice.	Different population: Acute Kidney Injury (AKI), not CKD.
24	Packham DK, 2015	Mesenchymal Precursor Cell Therapy for Diabetic Nephropathy: 24 Week Results from a Phase 2A Randomized Controlled Trial	Overlapping data with a study by Packham DK, 2016.
25	Bastug, 2014	Compare the effects of intravenous and intraperitoneal mesenchymal stem cell transplantation on ultrafiltration failure in a rat model of chronic peritoneal dialysis.	1) Comparison of two administration routes for MSCs (IV vs. IP), but no clarity on which MSC group was analyzed. 2) Outcome samples were taken from peritoneal tissue, not kidney tissue.
26	Bastug F, 2013	Mesenchymal stem cell transplantation may provide a new therapy for ultrafiltration failure in chronic peritoneal dialysis.	1) Different population: Peritoneal fibrosis. 2) Different outcome: outcomes derived from peritoneal membrane, not kidney tissue.
27	Reinders, 2013	Bone marrow-derived mesenchymal stromal cells from patients with end-stage renal disease are suitable for autologous therapy	1) Different population: ESRD patients and healthy controls. 2) No intervention. 3) Study aim was to characterize MSCs from ESRD patients vs. healthy controls, not to administer MSC therapy.
28	El-Ansary, 2012	Mesenchymal stem cells are a rescue approach for recovery of deteriorating kidney function.	All groups received MSCs, the study compared the administration of MSCs among different groups of CKD etiologies.

29	Kim JH, 2012	Human adipose tissue-derived mesenchymal stem cells protect kidneys from cisplatin nephrotoxicity in rats.	Different population: AKI.
30	Tulpar, 2012	Modulation of inflammation by mesenchymal stem cell transplantation in peritoneal dialysis in rats.	1) Different population: Peritoneal fibrosis. 2) Wrong outcome: outcomes derived from peritoneal membrane, not kidney tissue.
31	Asanuma, 2011	Arterially Delivered Mesenchymal Stem Cells Prevent Obstruction-Induced Renal Fibrosis	Different outcome, only histological markers were reported such as tubular atrophy, collagen deposition, α-SMA, and vimentin.
32	Unknown, 2011 (NL-OMON34665)	Application of mesenchymal stem cells in patients with end-stage renal disease	Withdrawn status from International Clinical Trials Registry Platform (ICTRP) and no result has been issued.
33	Ezquer F, 2009	Endovenous administration of bone-marrow-derived multipotent mesenchymal stromal cells prevents renal failure in diabetic mice.	Only urinary albumin was reported, and the measurement unit was not specified.

Supplementary figures

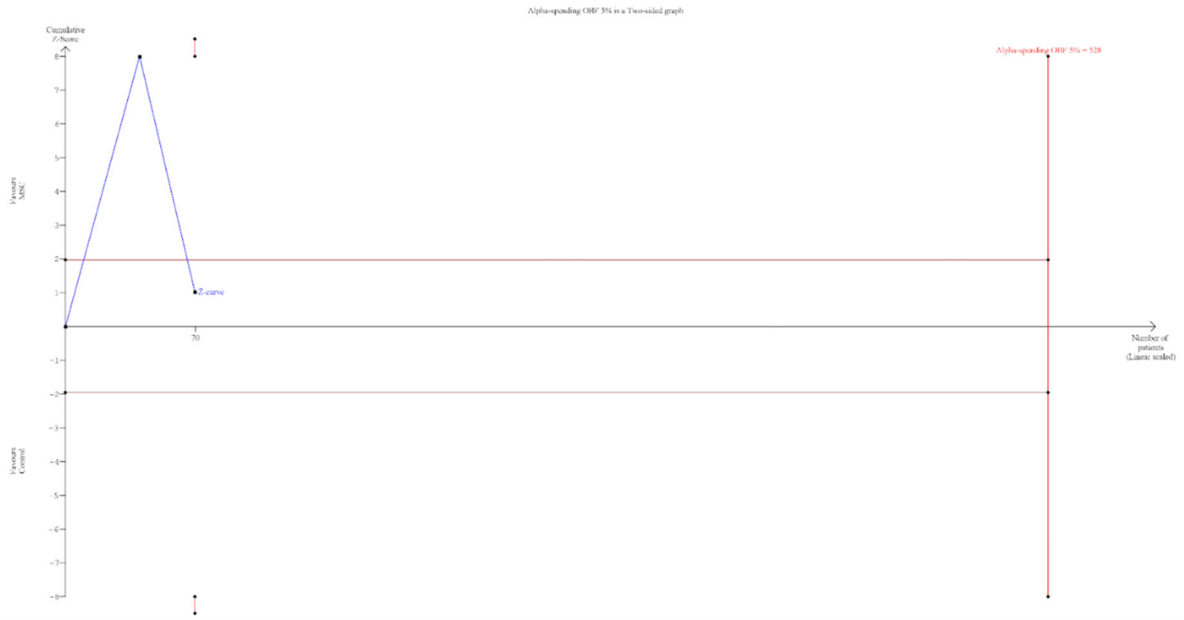

Figure S1. TSA Analysis for TNF-α in human RCTs. With parameters set at alpha 5%, beta 20% (power 80%), and the calculated diversity-adjusted required information size, the cumulative Z-curve did not cross the LDOF monitoring boundaries for benefit or harm, and did not approach the futility boundary. The final Z-value remained far from the required information size. The TSA-adjusted confidence interval crosses the line of no effect.

Study or Subgroup	MSC Mean	MSC SD	MSC Total	Control Mean	Control SD	Control Total	Weight	Mean Difference IV, Random, 95% CI	Year
**3.1.1 ≤ 2 weeks**									
Liu B, 2018	0.33	0.03	8	0.43	0.03	8	3.2%	-0.10 [-0.13, -0.07]	2018
Chen L, 2020	1.29	0.2236	5	1.68	0.313	5	2.1%	-0.39 [-0.73, -0.05]	2020
Liu B, 2020	0.37	0.02	8	0.45	0.02	8	3.2%	-0.08 [-0.10, -0.06]	2020
Liu L, 2021	0.4033	0.0789	6	0.5046	0.0867	6	3.1%	-0.10 [-0.20, -0.01]	2021
Rafiee Z, 2022	0.76	0.4808	8	1.02	0.2828	8	1.9%	-0.26 [-0.65, 0.13]	2022
**Subtotal (95% CI)**			**35**			**35**	**13.6%**	**-0.09 [-0.12, -0.07]**	
Heterogeneity: Tau² = 0.00; Chi² = 5.20, df = 4 (P = 0.27); I² = 23%									
Test for overall effect: Z = 7.42 (P < 0.00001)									
**3.1.2 ≤ 4 weeks**									
Xia C, 2021b	0.2036	0.1131	10	0.5994	0.3054	10	2.7%	-0.40 [-0.60, -0.19]	
Abdel Aziz, 2014	0.55	0.05	20	0.96	0.06	20	3.2%	-0.41 [-0.44, -0.38]	2014
Moghadasali, 2015	0.9	0.1	3	0.8	0.1	3	2.9%	0.10 [-0.06, 0.26]	2015
Anan, 2016	0.8	0.2236	5	1.5	0.2828	8	2.4%	-0.70 [-0.98, -0.42]	2016
Pan, 2017	1.28	0.16	10	1.76	0.14	10	3.0%	-0.48 [-0.61, -0.35]	2017
Xiang E, 2020	1.24	0.1118	5	1.67	0.3578	5	2.2%	-0.43 [-0.76, -0.10]	2020
Yiu, 2020	0.33	0.03	8	0.41	0.05	8	3.2%	-0.08 [-0.12, -0.04]	2020
Yea JH, 2021	1.53	0.02	5	1.7	0.08	5	3.1%	-0.17 [-0.24, -0.10]	2021
Akan E, 2021	0.5	0.2449	6	0.69	0.2449	6	2.4%	-0.19 [-0.47, 0.09]	2021
Xia C, 2021a	0.2036	0.1018	10	0.3969	0.1402	10	3.1%	-0.19 [-0.30, -0.09]	2021
Miyasaki DM, 2022	1.2	0.4	9	1.1	0.2	9	2.3%	0.10 [-0.19, 0.39]	2022
**Subtotal (95% CI)**			**91**			**94**	**30.5%**	**-0.25 [-0.38, -0.12]**	
Heterogeneity: Tau² = 0.04; Chi² = 206.12, df = 10 (P < 0.00001); I² = 95%									
Test for overall effect: Z = 3.82 (P = 0.0001)									
**3.1.3 ≤ 8 weeks**									
Villanueva, 2011	0.4	0.1	7	1	0.3	7	2.6%	-0.60 [-0.83, -0.37]	2011
Jia, 2016	1.38	0.17	12	2.07	0.47	12	2.4%	-0.69 [-0.97, -0.41]	2016
Anan, 2016	0.8	0.2236	5	1.5	0.2828	8	2.4%	-0.70 [-0.98, -0.42]	2016
Rashed, 2018	0.6	0.1	10	1.65	0.29	10	2.8%	-1.05 [-1.24, -0.86]	2018
Cetinkaya, 2019	0.4	0.1	6	0.8	0.06	6	3.1%	-0.40 [-0.49, -0.31]	2019
Xiang E, 2020	1.24	0.1118	5	1.67	0.3578	5	2.2%	-0.43 [-0.76, -0.10]	2020
Sheu JJ, 2020	0.89	0.07	6	1.27	0.08	6	3.1%	-0.38 [-0.47, -0.29]	2020
Lin L, 2020	0.68	0.18	10	4.36	2.23	10	0.3%	-3.68 [-5.07, -2.29]	2020
Wang S, 2021	1.11	0.1	9	1.27	0.14	9	3.0%	-0.16 [-0.27, -0.05]	2021
Alasmari, 2022	2.1	0.21	8	4.8	0.18	8	2.8%	-2.70 [-2.89, -2.51]	2022
Zhao X, 2024	20	3.9686	7	25	4.4978	7	0.0%	-5.00 [-9.44, -0.56]	2024
**Subtotal (95% CI)**			**85**			**88**	**24.6%**	**-0.97 [-1.40, -0.54]**	
Heterogeneity: Tau² = 0.45; Chi² = 607.34, df = 10 (P < 0.00001); I² = 98%									
Test for overall effect: Z = 4.44 (P < 0.00001)									
**3.1.4 ≤ 12 weeks**									
Ninichuk, 2006	0.82	1.3598	10	0.77	1.3598	10	0.4%	0.05 [-1.14, 1.24]	2006
Ma, 2013	0.58	0.2694	6	0.59	0.196	6	2.4%	-0.01 [-0.28, 0.26]	2013
Pan XH, 2014	0.4	0.05	6	0.5	0.05	6	3.2%	-0.10 [-0.16, -0.04]	2014
Caldas, 2015	1.02	0.19	10	1.34	0.42	10	2.3%	-0.32 [-0.61, -0.03]	2015
Yue Y, 2022	0.66	0.035	6	0.85	0.06	6	3.2%	-0.19 [-0.25, -0.13]	2022
Morsy S, 2022	0.48	0.08	6	0.74	0.09	6	3.1%	-0.26 [-0.36, -0.16]	2022
Alasmari WA, 2022	1.7	0.4	7	4.2	0.5	7	1.6%	-2.50 [-2.97, -2.03]	2022
Ni, 2023	0.29	0.07	6	0.36	0.05	6	3.2%	-0.07 [-0.14, -0.00]	2023
Shati, 2023	1.23	0.2324	15	1.43	0.3486	15	2.7%	-0.20 [-0.41, 0.01]	2023
**Subtotal (95% CI)**			**72**			**72**	**22.1%**	**-0.30 [-0.45, -0.15]**	
Heterogeneity: Tau² = 0.04; Chi² = 112.16, df = 8 (P < 0.00001); I² = 93%									
Test for overall effect: Z = 3.97 (P < 0.0001)									
**3.1.5 > 12 weeks**									
Caldas, 2008	0.95	0.1	5	1.31	0.5	5	1.7%	-0.36 [-0.81, 0.09]	2008
Choi S, 2009	0.43	0.11	6	0.46	0.09	3	3.0%	-0.03 [-0.16, 0.10]	2009
Jiao YQ, 2011	0.43	0.426	15	0.46	0.3486	15	2.4%	-0.03 [-0.31, 0.25]	2011
An, 2019	1.12	0.2939	6	1.25	0.22	4	2.2%	-0.13 [-0.45, 0.19]	2019
**Subtotal (95% CI)**			**32**			**27**	**9.2%**	**-0.06 [-0.17, 0.05]**	
Heterogeneity: Tau² = 0.00; Chi² = 2.15, df = 3 (P = 0.54); I² = 0%									
Test for overall effect: Z = 1.10 (P = 0.27)									
**Total (95% CI)**			**315**			**316**	**100.0%**	**-0.39 [-0.47, -0.30]**	
Heterogeneity: Tau² = 0.06; Chi² = 1360.58, df = 39 (P < 0.00001); I² = 97%									
Test for overall effect: Z = 9.01 (P < 0.00001)									
Test for subgroup differences: Chi² = 29.10, df = 4 (P < 0.00001), I² = 86.3%									

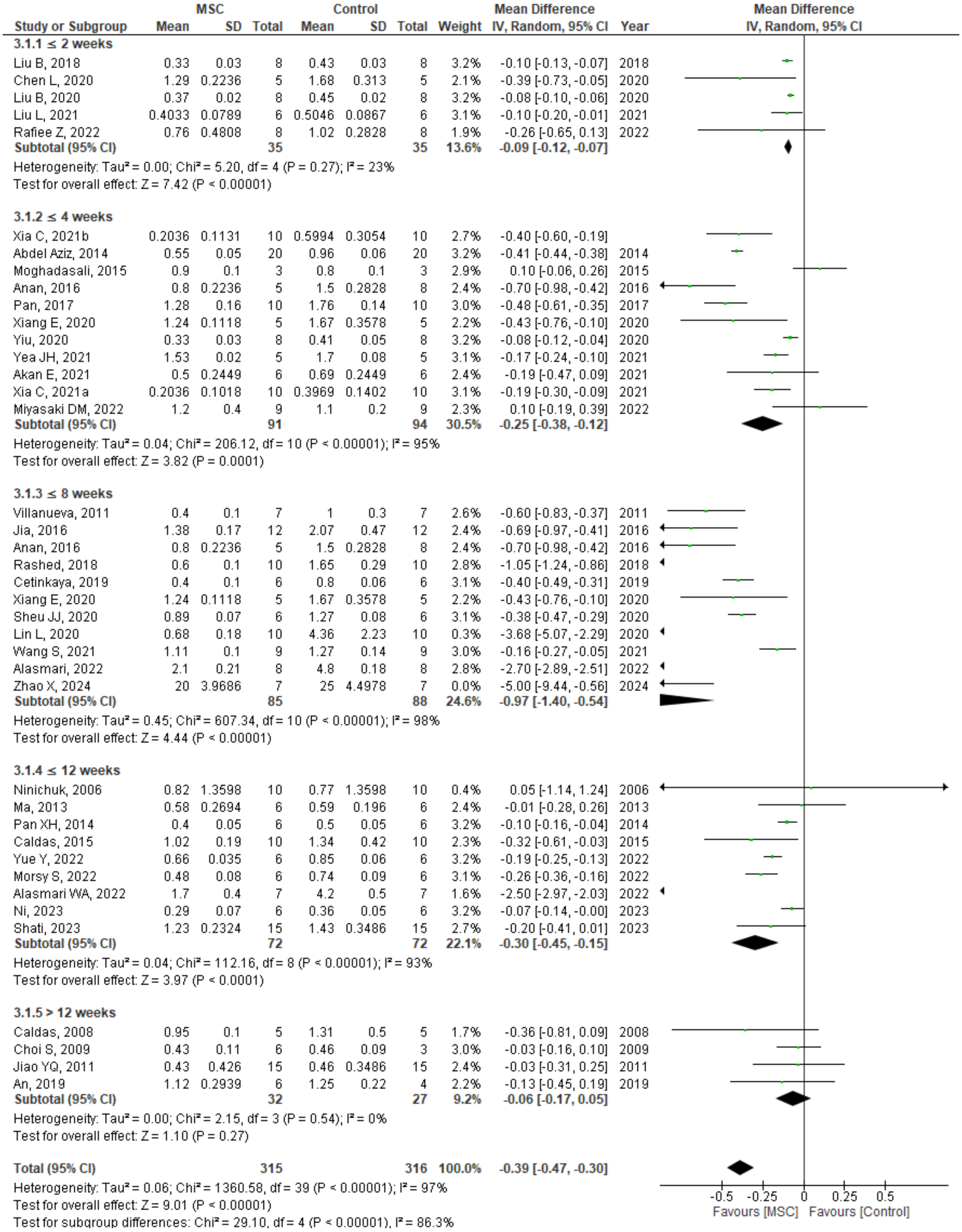

Figure S2. Forest plot of the effects of MSC treatment on serum creatinine (mg/dL) in animal studies with subgroup analysis based on duration of follow-up (0 to ≤ 2 weeks; > 2 to ≤ 4 weeks; > 4 to ≤ 8 weeks; > 8 to ≤ 12 weeks; and > 12 weeks). Figure was generated using Review Manager (RevMan) version 5.4, The Cochrane Collaboration.

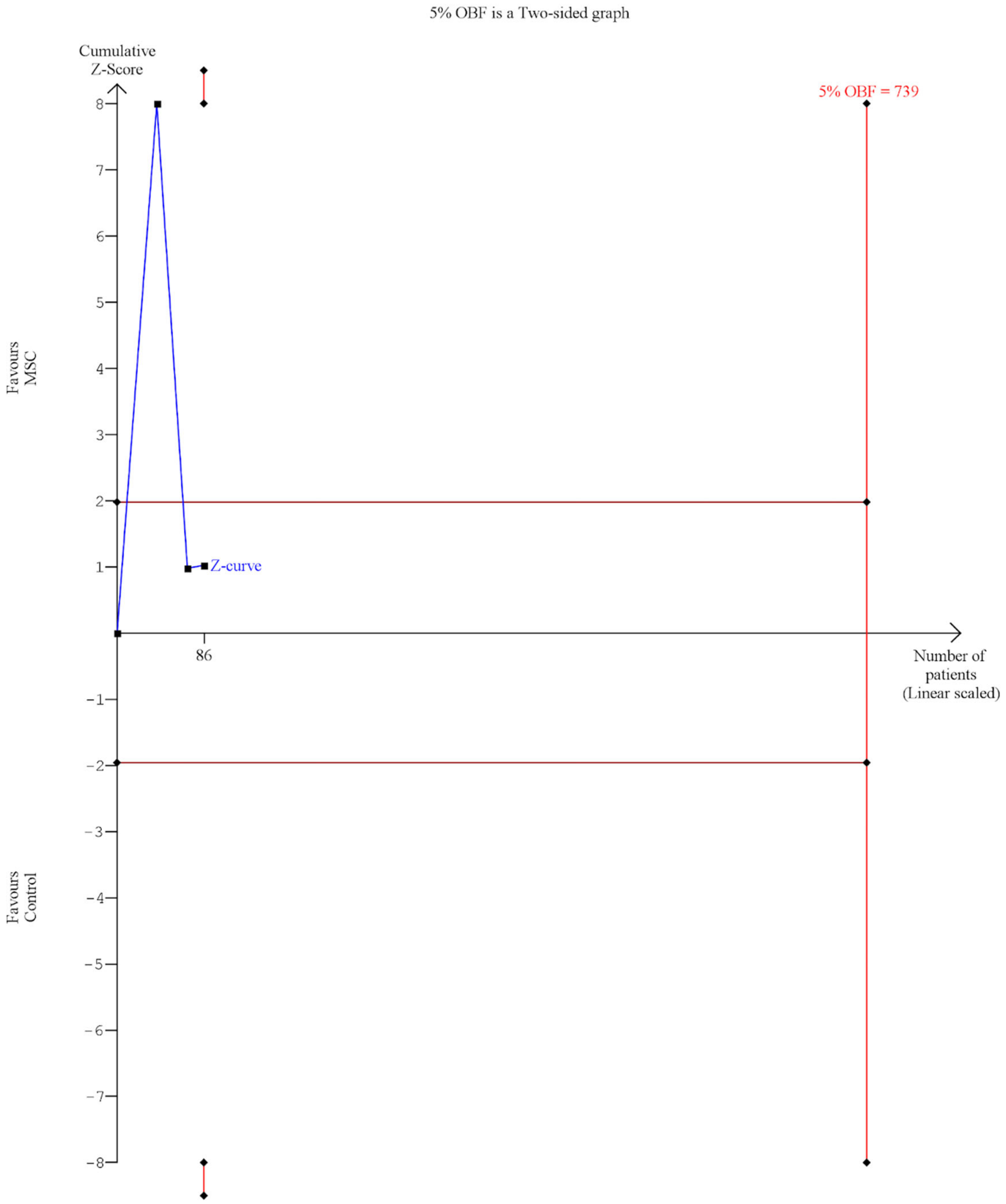

Figure S3. TSA Analysis for serum creatinine. Using the chosen parameters (alpha 5%, beta 20% [power 80%], diversity-adjusted required information size as calculated), the cumulative Z-curve did not cross the Lan-DeMets O'Brien-Fleming (LDOF) monitoring boundary for benefit, nor the futility or harm boundaries. The curve also did not reach the required information size, indicating insufficient evidence for firm conclusions. The TSA-adjusted confidence interval spans both potential benefit and no effect.

Study or Subgroup	MSC Mean	MSC SD	MSC Total	Control Mean	Control SD	Control Total	Weight	Mean Difference IV, Random, 95% CI	Year
**20.1.1 Umbilical cord**									
Ma, 2013	0.58	0.2694	6	0.59	0.196	6	2.5%	-0.01 [-0.28, 0.26]	2013
Liu B, 2018	0.33	0.03	8	0.43	0.03	8	3.4%	-0.10 [-0.13, -0.07]	2018
An, 2019	1.12	0.2939	6	1.25	0.22	4	2.3%	-0.13 [-0.45, 0.19]	2019
Chen L, 2020	1.29	0.2236	5	1.68	0.313	5	2.2%	-0.39 [-0.73, -0.05]	2020
Liu B, 2020	0.37	0.02	8	0.45	0.02	8	3.4%	-0.08 [-0.10, -0.06]	2020
Xiang E, 2020	1.24	0.1118	5	1.67	0.3578	5	2.3%	-0.43 [-0.76, -0.10]	2020
Yiu, 2020	0.33	0.03	8	0.41	0.05	8	3.4%	-0.08 [-0.12, -0.04]	2020
Miyasaki DM, 2022	1.2	0.4	9	1.1	0.2	9	2.4%	0.10 [-0.19, 0.39]	2022
Yue Y, 2022	0.66	0.035	6	0.85	0.06	6	3.3%	-0.19 [-0.25, -0.13]	2022
**Subtotal (95% CI)**			**61**			**59**	**25.2%**	**-0.11 [-0.15, -0.07]**	
Heterogeneity: Tau² = 0.00; Chi² = 23.13, df = 8 (P = 0.003); I² = 65%									
Test for overall effect: Z = 5.56 (P < 0.00001)									
**20.1.2 Bone marrow**									
Xia C, 2021b	0.2036	0.1131	10	0.5994	0.3054	10	2.8%	-0.40 [-0.60, -0.19]	
Ninichuk, 2006	0.82	1.3598	10	0.77	1.3598	10	0.4%	0.05 [-1.14, 1.24]	2006
Caldas, 2008	0.95	0.1	5	1.31	0.5	5	1.8%	-0.36 [-0.81, 0.09]	2008
Choi S, 2009	0.43	0.11	6	0.46	0.09	3	3.1%	-0.03 [-0.16, 0.10]	2009
Villanueva, 2011	0.4	0.1	7	1	0.3	7	2.7%	-0.60 [-0.83, -0.37]	2011
Abdel Aziz, 2014	0.55	0.05	20	0.96	0.06	20	3.4%	-0.41 [-0.44, -0.38]	2014
Pan XH, 2014	0.4	0.05	6	0.5	0.05	6	3.3%	-0.10 [-0.16, -0.04]	2014
Caldas, 2015	1.02	0.19	10	1.34	0.42	10	2.5%	-0.32 [-0.61, -0.03]	2015
Moghadasali, 2015	0.9	0.1	3	0.8	0.1	3	3.0%	0.10 [-0.06, 0.26]	2015
Anan, 2016	0.8	0.2236	5	1.5	0.2828	8	2.5%	-0.70 [-0.98, -0.42]	2016
Jia, 2016	1.38	0.17	12	2.07	0.47	12	2.5%	-0.69 [-0.97, -0.41]	2016
Rashed, 2018	0.6	0.1	10	1.65	0.29	10	2.9%	-1.05 [-1.24, -0.86]	2018
Lin L, 2020	0.68	0.18	10	4.36	2.23	10	0.3%	-3.68 [-5.07, -2.29]	2020
Xia C, 2021a	0.2036	0.1018	10	0.3969	0.1402	10	3.2%	-0.19 [-0.30, -0.09]	2021
Wang S, 2021	1.11	0.1	9	1.27	0.14	9	3.2%	-0.16 [-0.27, -0.05]	2021
Alasmari WA, 2022	1.7	0.4	7	4.2	0.5	7	1.7%	-2.50 [-2.97, -2.03]	2022
Alasmari, 2022	2.1	0.21	8	4.8	0.18	8	2.9%	-2.70 [-2.89, -2.51]	2022
Morsy S, 2022	0.48	0.08	6	0.74	0.09	6	3.2%	-0.26 [-0.36, -0.16]	2022
Shati, 2023	1.23	0.2324	15	1.43	0.3486	15	2.8%	-0.20 [-0.41, 0.01]	2023
**Subtotal (95% CI)**			**169**			**169**	**48.3%**	**-0.64 [-0.85, -0.42]**	
Heterogeneity: Tau² = 0.19; Chi² = 910.66, df = 18 (P < 0.00001); I² = 98%									
Test for overall effect: Z = 5.87 (P < 0.00001)									
**20.1.3 Amnion**									
Cetinkaya, 2019	0.4	0.1	6	0.8	0.06	6	3.3%	-0.40 [-0.49, -0.31]	2019
Akan E, 2021	0.5	0.2449	6	0.69	0.2449	6	2.5%	-0.19 [-0.47, 0.09]	2021
Ni, 2023	0.29	0.07	6	0.36	0.05	6	3.3%	-0.07 [-0.14, -0.00]	2023
**Subtotal (95% CI)**			**18**			**18**	**9.1%**	**-0.22 [-0.48, 0.04]**	
Heterogeneity: Tau² = 0.05; Chi² = 31.11, df = 2 (P < 0.00001); I² = 94%									
Test for overall effect: Z = 1.69 (P = 0.09)									
**20.1.4 Adipose**									
Yea JH, 2021	1.53	0.02	5	1.7	0.08	5	3.3%	-0.17 [-0.24, -0.10]	2021
**Subtotal (95% CI)**			**5**			**5**	**3.3%**	**-0.17 [-0.24, -0.10]**	
Heterogeneity: Not applicable									
Test for overall effect: Z = 4.61 (P < 0.00001)									
**20.1.5 Others**									
Jiao YQ, 2011	0.43	0.426	15	0.46	0.3486	15	2.5%	-0.03 [-0.31, 0.25]	2011
Pan, 2017	1.28	0.16	10	1.76	0.14	10	3.1%	-0.48 [-0.61, -0.35]	2017
Sheu JJ, 2020	0.89	0.07	6	1.27	0.08	6	3.3%	-0.38 [-0.47, -0.29]	2020
Liu L, 2021	0.4033	0.0789	6	0.5046	0.0867	6	3.3%	-0.10 [-0.20, -0.01]	2021
Rafiee Z, 2022	0.76	0.4808	8	1.02	0.2828	8	2.0%	-0.26 [-0.65, 0.13]	2022
**Subtotal (95% CI)**			**45**			**45**	**14.2%**	**-0.26 [-0.44, -0.09]**	
Heterogeneity: Tau² = 0.03; Chi² = 31.17, df = 4 (P < 0.00001); I² = 87%									
Test for overall effect: Z = 2.89 (P = 0.004)									
**Total (95% CI)**			**298**			**296**	**100.0%**	**-0.38 [-0.46, -0.29]**	
Heterogeneity: Tau² = 0.06; Chi² = 1339.53, df = 36 (P < 0.00001); I² = 97%									
Test for overall effect: Z = 8.59 (P < 0.00001)									
Test for subgroup differences: Chi² = 26.15, df = 4 (P < 0.0001), I² = 84.7%									

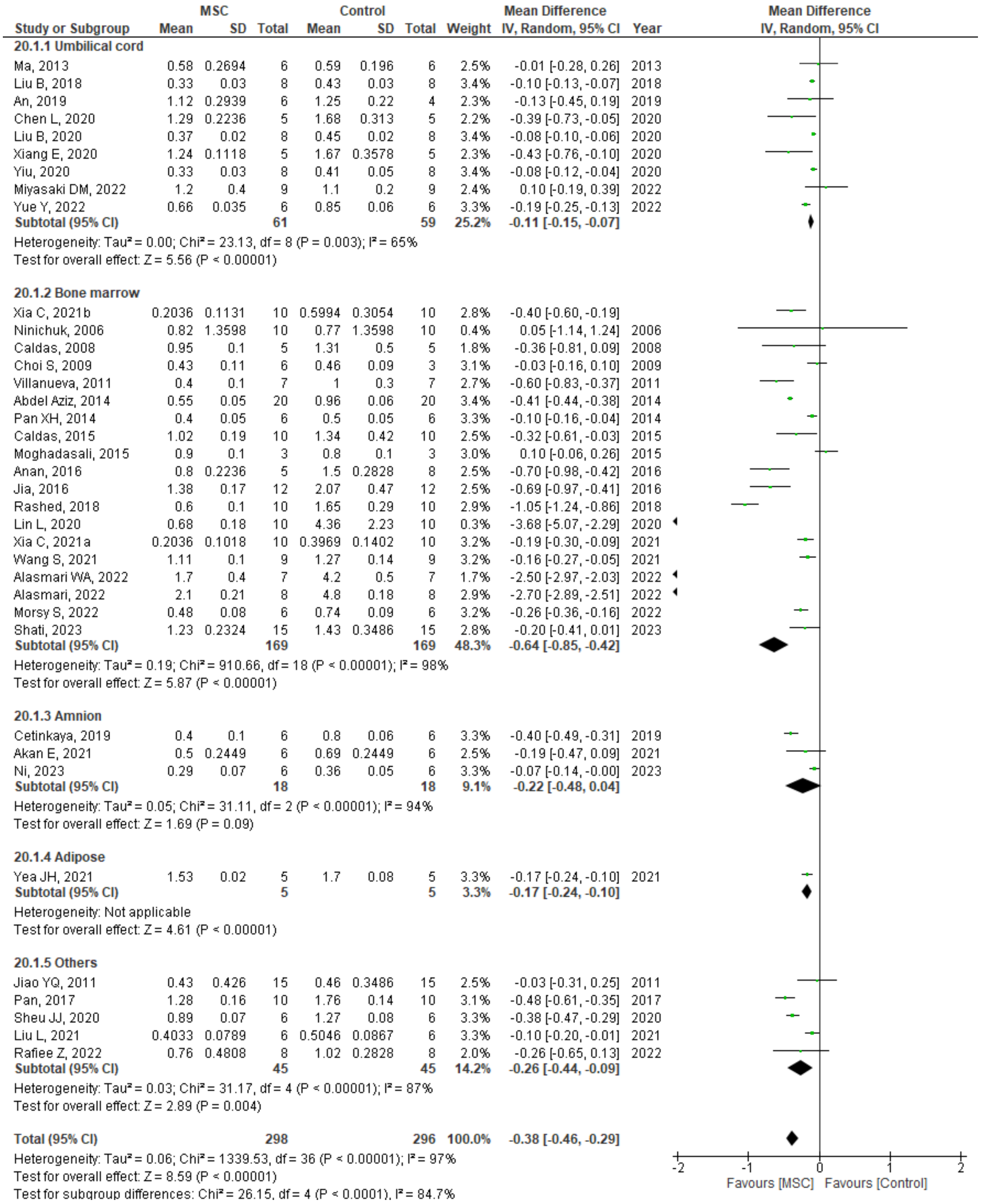

Figure S4. Forest plot of the effects of MSC treatment on serum creatinine (mg/dL) in animal studies with subgroup analysis based on source of MSC (umbilical cord, bone marrow, amnion, adipose tissue, and others). Figure was generated using Review Manager (RevMan) version 5.4, The Cochrane Collaboration.

Study or Subgroup	MSC Mean	MSC SD	MSC Total	Control Mean	Control SD	Control Total	Weight	Mean Difference IV, Random, 95% CI	Year
**21.1.1 Whole cell**									
Xia C, 2021b	0.2036	0.1131	10	0.5994	0.3054	10	2.8%	-0.40 [-0.60, -0.19]	
Ninichuk, 2006	0.82	1.3598	10	0.77	1.3598	10	0.5%	0.05 [-1.14, 1.24]	2006
Caldas, 2008	0.95	0.1	5	1.31	0.5	5	1.8%	-0.36 [-0.81, 0.09]	2008
Choi S, 2009	0.43	0.11	6	0.46	0.09	3	3.1%	-0.03 [-0.16, 0.10]	2009
Jiao YQ, 2011	0.4	0.1	7	1	0.3	7	2.7%	-0.60 [-0.83, -0.37]	2011
Villanueva, 2011	0.4	0.1	7	1	0.3	7	2.7%	-0.60 [-0.83, -0.37]	2011
Ma, 2013	0.58	0.2694	6	0.59	0.196	6	2.5%	-0.01 [-0.28, 0.26]	2013
Abdel Aziz, 2014	0.55	0.05	20	0.96	0.06	20	3.4%	-0.41 [-0.44, -0.38]	2014
Pan XH, 2014	0.4	0.05	6	0.5	0.05	6	3.3%	-0.10 [-0.16, -0.04]	2014
Moghadasali, 2015	0.9	0.1	3	0.8	0.1	3	3.0%	0.10 [-0.06, 0.26]	2015
Caldas, 2015	1.02	0.19	10	1.34	0.42	10	2.5%	-0.32 [-0.61, -0.03]	2015
Jia, 2016	1.38	0.17	12	2.07	0.47	12	2.5%	-0.69 [-0.97, -0.41]	2016
Anan, 2016	0.8	0.2236	5	1.5	0.2828	8	2.5%	-0.70 [-0.98, -0.42]	2016
Pan, 2017	1.28	0.16	10	1.76	0.14	10	3.1%	-0.48 [-0.61, -0.35]	2017
Liu B, 2018	0.33	0.03	8	0.43	0.03	8	3.4%	-0.10 [-0.13, -0.07]	2018
Rashed, 2018	0.6	0.1	10	1.65	0.29	10	2.9%	-1.05 [-1.24, -0.86]	2018
An, 2019	1.12	0.2939	6	1.25	0.22	4	2.3%	-0.13 [-0.45, 0.19]	2019
Cetinkaya, 2019	0.4	0.1	6	0.8	0.06	6	3.2%	-0.40 [-0.49, -0.31]	2019
Lin L, 2020	0.68	0.18	10	4.36	2.23	10	0.3%	-3.68 [-5.07, -2.29]	2020
Sheu JJ, 2020	0.89	0.07	6	1.27	0.08	6	3.3%	-0.38 [-0.47, -0.29]	2020
Chen L, 2020	1.29	0.2236	5	1.68	0.313	5	2.2%	-0.39 [-0.73, -0.05]	2020
Yiu, 2020	0.33	0.03	8	0.41	0.05	8	3.3%	-0.08 [-0.12, -0.04]	2020
Akan E, 2021	0.5	0.2449	6	0.69	0.2449	6	2.5%	-0.19 [-0.47, 0.09]	2021
Xia C, 2021a	0.2036	0.1018	10	0.3969	0.1402	10	3.2%	-0.19 [-0.30, -0.09]	2021
Morsy S, 2022	0.48	0.08	6	0.74	0.09	6	3.2%	-0.26 [-0.36, -0.16]	2022
Rafiee Z, 2022	0.76	0.4808	8	1.02	0.2828	8	2.0%	-0.26 [-0.65, 0.13]	2022
Yue Y, 2022	0.66	0.035	6	0.85	0.06	6	3.3%	-0.19 [-0.25, -0.13]	2022
Ni, 2023	0.29	0.07	6	0.36	0.05	6	3.3%	-0.07 [-0.14, -0.00]	2023
Shati, 2023	1.23	0.2324	15	1.43	0.3486	15	2.8%	-0.20 [-0.41, 0.01]	2023
**Subtotal (95% CI)**			**233**			**231**	**77.7%**	**-0.31 [-0.38, -0.23]**	
Heterogeneity: Tau² = 0.03; Chi² = 476.80, df = 28 (P < 0.00001); I² = 94%									
Test for overall effect: Z = 7.86 (P < 0.00001)									
**21.1.2 Exosome**									
Liu B, 2020	0.37	0.02	8	0.45	0.02	8	3.4%	-0.08 [-0.10, -0.06]	2020
Xiang E, 2020	1.24	0.1118	5	1.67	0.3578	5	2.3%	-0.43 [-0.76, -0.10]	2020
Liu L, 2021	0.4033	0.0789	6	0.5046	0.0867	6	3.2%	-0.10 [-0.20, -0.01]	2021
Wang S, 2021	1.11	0.1	9	1.27	0.14	9	3.2%	-0.16 [-0.27, -0.05]	2021
Yea JH, 2021	1.53	0.02	5	1.7	0.08	5	3.3%	-0.17 [-0.24, -0.10]	2021
Miyasaki DM, 2022	1.2	0.4	9	1.1	0.2	9	2.4%	0.10 [-0.19, 0.39]	2022
Alasmari, 2022	2.1	0.21	8	4.8	0.18	8	2.9%	-2.70 [-2.89, -2.51]	2022
Alasmari WA, 2022	1.7	0.4	7	4.2	0.5	7	1.7%	-2.50 [-2.97, -2.03]	2022
**Subtotal (95% CI)**			**57**			**57**	**22.3%**	**-0.72 [-1.09, -0.35]**	
Heterogeneity: Tau² = 0.27; Chi² = 816.68, df = 7 (P < 0.00001); I² = 99%									
Test for overall effect: Z = 3.84 (P = 0.0001)									
**Total (95% CI)**			**290**			**288**	**100.0%**	**-0.39 [-0.48, -0.30]**	
Heterogeneity: Tau² = 0.06; Chi² = 1351.56, df = 36 (P < 0.00001); I² = 97%									
Test for overall effect: Z = 8.91 (P < 0.00001)									
Test for subgroup differences: Chi² = 4.63, df = 1 (P = 0.03), I² = 78.4%									

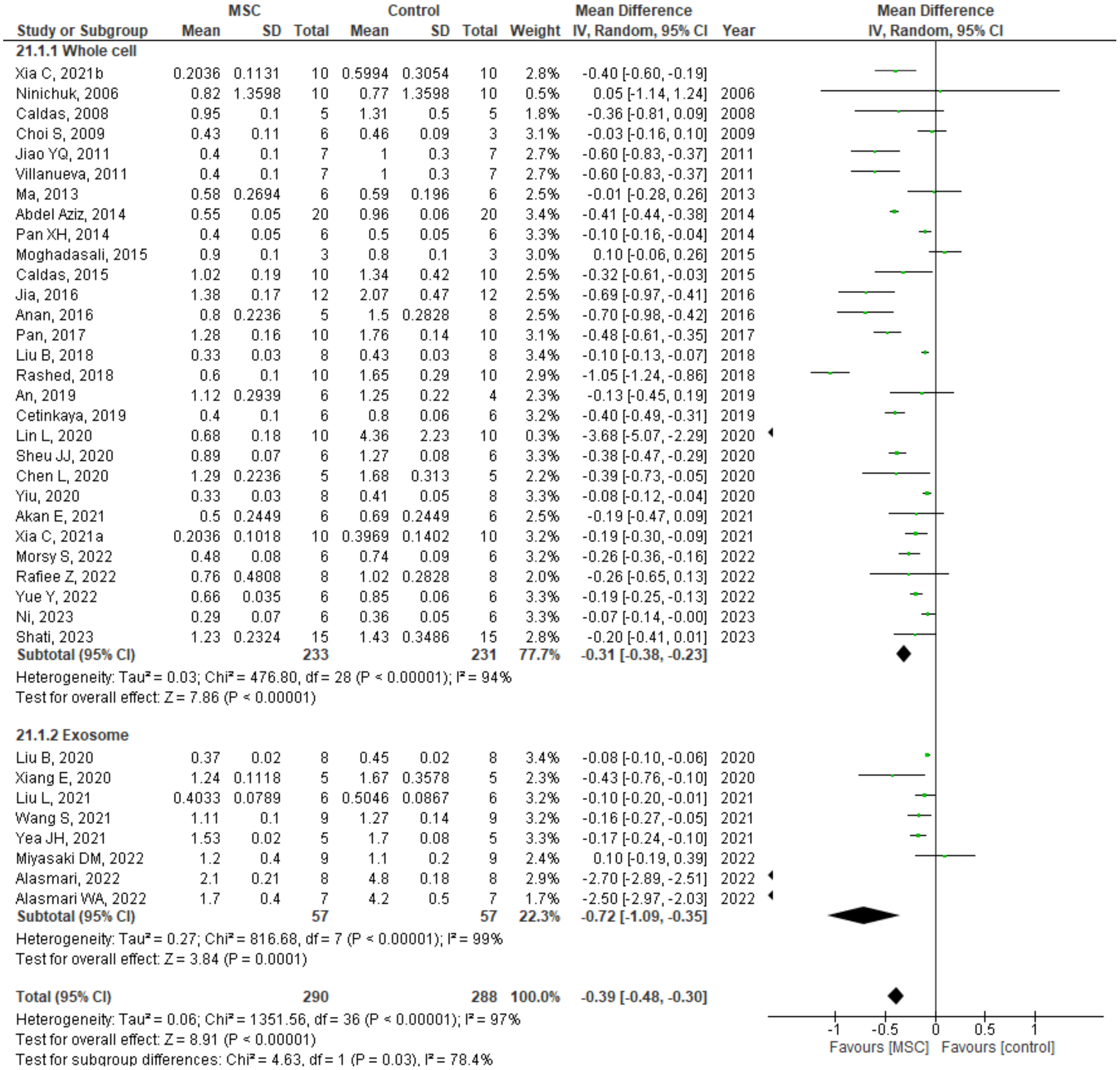

Figure S5. Forest plot of the effects of MSC treatment on serum creatinine (mg/dL) in animal studies with subgroup analysis based on MSC-derived product (whole cell and exosome). Figure was generated using Review Manager (RevMan) version 5.4, The Cochrane Collaboration.

Study or Subgroup	MSC Mean	MSC SD	MSC Total	Control Mean	Control SD	Control Total	Weight	Mean Difference IV, Random, 95% CI	Year
**5.1.1 ≤ 2 weeks**									
Liu B, 2018	33.6	5.6	8	47.61	5.6	8	3.5%	-14.01 [-19.50, -8.52]	2018
Chen L, 2020	21.48	1.7441	5	24.75	1.6994	5	3.7%	-3.27 [-5.40, -1.14]	2020
Liu B, 2020	33.6	5.6	8	47.61	5.6	8	3.5%	-14.01 [-19.50, -8.52]	2020
Liu L, 2021	22.5	6.6626	6	33.56	7.9853	6	3.3%	-11.06 [-19.38, -2.74]	2021
Rafiee Z, 2022	22.86	0.78	8	34	1.5	8	3.7%	-11.14 [-12.31, -9.97]	2022
**Subtotal (95% CI)**			**35**			**35**	**17.8%**	**-10.33 [-15.06, -5.60]**	
Heterogeneity: Tau^2 = 23.29; Chi^2 = 45.49, df = 4 (P < 0.00001); I^2 = 91%									
Test for overall effect: Z = 4.28 (P < 0.0001)									
**5.1.2 ≤ 4 weeks**									
Xia C, 2021b	16.8	5.04	10	43.69	22.4	10	2.6%	-26.89 [-41.12, -12.66]	
Moghadasali, 2015	26.8	5.16	3	22.58	9.44	3	2.9%	4.22 [-7.95, 16.39]	2015
Pan, 2017	19.46	4.51	10	30.27	3.72	10	3.7%	-10.81 [-14.43, -7.19]	2017
Yiu, 2020	70	17	8	109	6	8	2.8%	-39.00 [-51.49, -26.51]	2020
Xia C, 2021a	16.8	5.6	10	25.2	5.6	10	3.6%	-8.40 [-13.31, -3.49]	2021
Yea JH, 2021	32.2	1.2	5	51.9	1.8	5	3.7%	-19.70 [-21.60, -17.80]	2021
Akan E, 2021	44.76	146.9694	6	50	14.6969	6	0.1%	-5.24 [-123.42, 112.94]	2021
Morsy S, 2022	38.4	4.1	6	80.4	6.11	6	3.5%	-42.00 [-47.89, -36.11]	2022
Ni, 2023	33.91	3	6	104.42	10.61	6	3.2%	-70.51 [-79.33, -61.69]	2023
Shati, 2023	153.24	10.82	15	211.53	13.83	15	3.2%	-58.29 [-67.18, -49.40]	2023
**Subtotal (95% CI)**			**79**			**79**	**29.4%**	**-29.86 [-41.22, -18.49]**	
Heterogeneity: Tau^2 = 284.86; Chi^2 = 320.18, df = 9 (P < 0.00001); I^2 = 97%									
Test for overall effect: Z = 5.15 (P < 0.00001)									
**5.1.3 ≤ 8 weeks**									
Anan, 2016	33	7.1	5	60	7.1	8	3.3%	-27.00 [-34.93, -19.07]	2016
Jia, 2016	31.45	4.06	12	39.9	3.44	12	3.7%	-8.45 [-11.46, -5.44]	2016
Rashed, 2018	62.23	3.9	10	84.06	9.49	10	3.5%	-21.83 [-28.19, -15.47]	2018
Cetinkaya, 2019	16	2	6	30	0.9	6	3.7%	-14.00 [-15.75, -12.25]	2019
Lin L, 2020	890	180	10	1,940	250	10	0.0%	-1050.00 [-1240.93, -859.07]	2020
Sheu JJ, 2020	44.8	1	6	61.57	3.53	6	3.7%	-16.77 [-19.71, -13.83]	2020
Xiang E, 2020	22.4	1.8783	5	25.2	2.5044	5	3.7%	-2.80 [-5.54, -0.06]	2020
Wang S, 2021	25.12	10.5	9	41	14.11	9	2.9%	-15.88 [-27.37, -4.39]	2021
Alasmari, 2022	48	4	7	78	9	7	3.4%	-30.00 [-37.30, -22.70]	2022
Zhao X, 2024	6.42	0.29	7	8.03	0.86	7	3.8%	-1.61 [-2.28, -0.94]	2024
**Subtotal (95% CI)**			**77**			**80**	**31.7%**	**-16.18 [-22.92, -9.45]**	
Heterogeneity: Tau^2 = 98.06; Chi^2 = 483.94, df = 9 (P < 0.00001); I^2 = 98%									
Test for overall effect: Z = 4.71 (P < 0.00001)									
**5.1.4 ≤ 12 weeks**									
Ninichuk, 2006	131.6	181.831	10	107	264.3664	10	0.0%	24.60 [-174.27, 223.47]	2006
Ma, 2013	29.65	7.22	6	25.37	4.9	6	3.4%	4.28 [-2.70, 11.26]	2013
Pan XH, 2014	35.28	3.64	6	45.36	7	6	3.5%	-10.08 [-16.39, -3.77]	2014
Alasmari WA, 2022	49.4	1.95	8	81.2	3.43	8	3.7%	-31.80 [-34.53, -29.07]	2022
Yue Y, 2022	46	3	6	101	15	6	2.9%	-55.00 [-67.24, -42.76]	2022
**Subtotal (95% CI)**			**36**			**36**	**13.5%**	**-22.25 [-42.33, -2.18]**	
Heterogeneity: Tau^2 = 408.11; Chi^2 = 135.73, df = 4 (P < 0.00001); I^2 = 97%									
Test for overall effect: Z = 2.17 (P = 0.03)									
**5.1.5 > 12 weeks**									
Jiao YQ, 2011	25.87	17.8157	15	30.74	22.3471	15	2.6%	-4.87 [-19.33, 9.59]	2011
Li Y, 2018	36.4	11.2	11	42	28	14	2.4%	-5.60 [-21.69, 10.49]	2018
An, 2019	28.73	8.9896	6	34.53	13.72	4	2.5%	-5.80 [-21.05, 9.45]	2019
**Subtotal (95% CI)**			**32**			**33**	**7.6%**	**-5.40 [-14.19, 3.39]**	
Heterogeneity: Tau^2 = 0.00; Chi^2 = 0.01, df = 2 (P = 1.00); I^2 = 0%									
Test for overall effect: Z = 1.20 (P = 0.23)									
**Total (95% CI)**			**259**			**263**	**100.0%**	**-19.27 [-23.51, -15.03]**	
Heterogeneity: Tau^2 = 124.70; Chi^2 = 1674.83, df = 32 (P < 0.00001); I^2 = 98%									
Test for overall effect: Z = 8.90 (P < 0.00001)									
Test for subgroup differences: Chi^2 = 14.22, df = 4 (P = 0.007), I^2 = 71.9%									

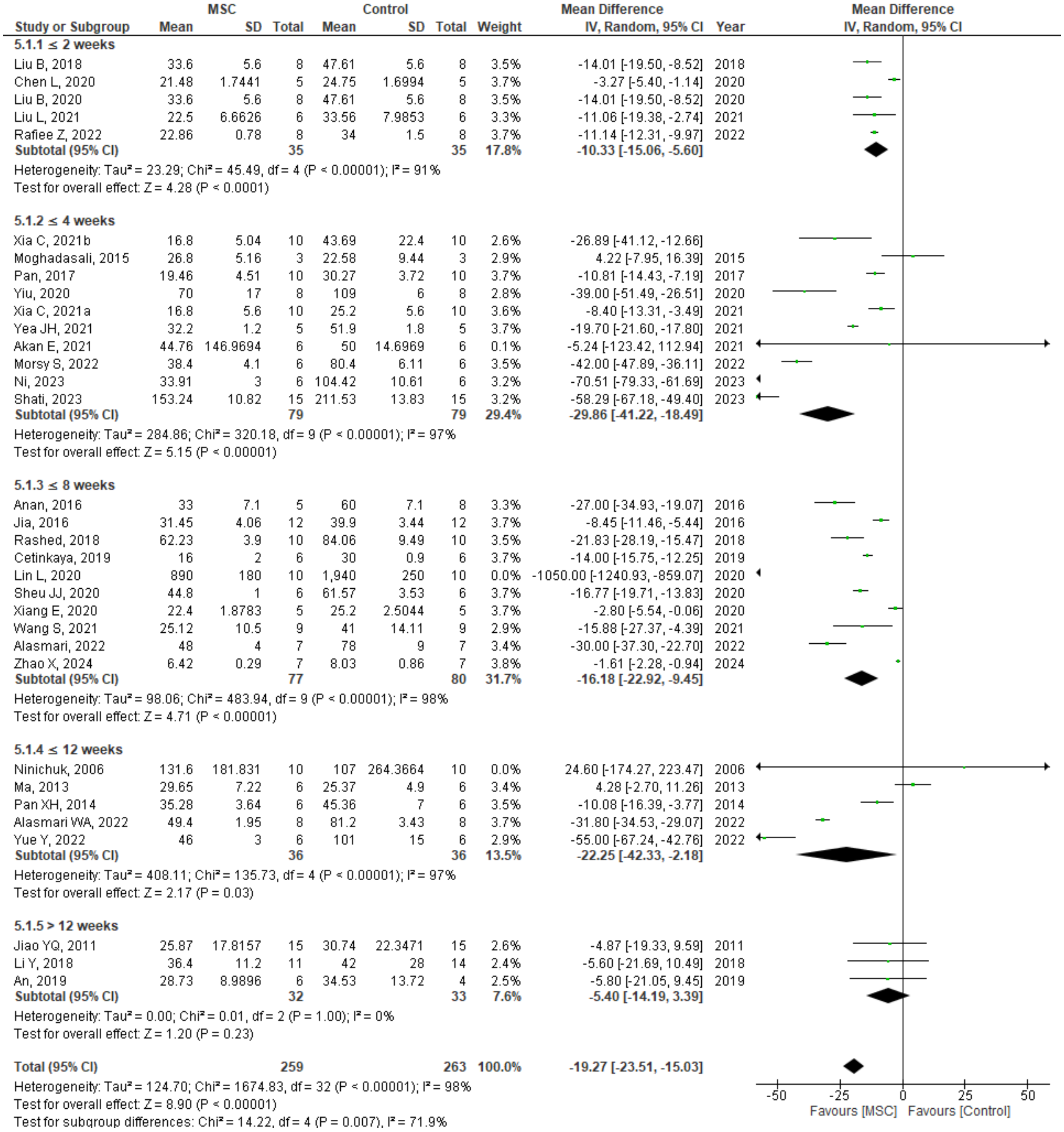

Figure S6. Forest plot of the effect of MSC treatment on BUN (mg/dL) in animal studies with subgroup analysis based on duration of follow-up (0 to ≤ 2 weeks; > 2 to ≤ 4 weeks; > 4 to ≤ 8 weeks; > 8 to ≤ 12 weeks; and > 12 weeks). Figure was generated using Review Manager (RevMan) version 5.4, The Cochrane Collaboration.

Study or Subgroup	MSC Mean	MSC SD	MSC Total	Control Mean	Control SD	Control Total	Weight	Mean Difference IV, Random, 95% CI	Year
**22.1.1 Umbilical cord**									
Ma, 2013	29.65	7.22	6	25.37	4.9	6	3.8%	4.28 [-2.70, 11.26]	2013
Liu B, 2018	33.6	5.6	8	47.61	5.6	8	4.0%	-14.01 [-19.50, -8.52]	2018
An, 2019	28.73	8.9896	6	34.53	13.72	4	2.7%	-5.80 [-21.05, 9.45]	2019
Chen L, 2020	21.48	1.7441	5	24.75	1.6994	5	4.3%	-3.27 [-5.40, -1.14]	2020
Liu B, 2020	33.6	5.6	8	47.61	5.6	8	4.0%	-14.01 [-19.50, -8.52]	2020
Xiang E, 2020	22.4	1.8783	5	25.2	2.5044	5	4.3%	-2.80 [-5.54, -0.06]	2020
Yiu, 2020	70	17	8	109	6	8	3.0%	-39.00 [-51.49, -26.51]	2020
Yue Y, 2022	46	3	6	101	15	6	3.1%	-55.00 [-67.24, -42.76]	2022
**Subtotal (95% CI)**			**52**			**50**	**29.2%**	**-14.64 [-22.17, -7.12]**	
Heterogeneity: Tau² = 99.67; Chi² = 124.34, df = 7 (P < 0.00001); I² = 94%									
Test for overall effect: Z = 3.81 (P = 0.0001)									
**22.1.2 Bone marrow**									
Xia C, 2021b	16.8	5.04	10	43.69	22.4	10	2.8%	-26.89 [-41.12, -12.66]	
Ninichuk, 2006	131.6	181.831	10	107	264.3664	10	0.0%	24.60 [-174.27, 223.47]	2006
Pan XH, 2014	35.28	3.64	6	45.36	7	6	3.9%	-10.08 [-16.39, -3.77]	2014
Moghadasali, 2015	26.8	5.16	3	22.58	9.44	3	3.1%	4.22 [-7.95, 16.39]	2015
Anan, 2016	33	7.1	5	60	7.1	8	3.7%	-27.00 [-34.93, -19.07]	2016
Jia, 2016	31.45	4.06	12	39.9	3.44	12	4.2%	-8.45 [-11.46, -5.44]	2016
Li Y, 2018	36.4	11.2	11	42	28	14	2.5%	-5.60 [-21.69, 10.49]	2018
Rashed, 2018	62.23	3.9	10	84.06	9.49	10	3.9%	-21.83 [-28.19, -15.47]	2018
Xia C, 2021a	16.8	5.6	10	25.2	5.6	10	4.1%	-8.40 [-13.31, -3.49]	2021
Wang S, 2021	25.12	10.5	9	41	14.11	9	3.2%	-15.88 [-27.37, -4.39]	2021
Alasmari, 2022	49.4	1.95	8	81.2	3.43	8	4.3%	-31.80 [-34.53, -29.07]	2022
Morsy S, 2022	38.4	4.1	6	80.4	6.11	0		Not estimable	2022
Shati, 2023	153.24	10.82	15	211.53	13.83	15	3.6%	-58.29 [-67.18, -49.40]	2023
**Subtotal (95% CI)**			**115**			**115**	**39.3%**	**-19.24 [-28.23, -10.24]**	
Heterogeneity: Tau² = 210.19; Chi² = 253.53, df = 11 (P < 0.00001); I² = 96%									
Test for overall effect: Z = 4.19 (P < 0.0001)									
**22.1.3 Amnion**									
Cetinkaya, 2019	16	2	6	30	0.9	6	4.3%	-14.00 [-15.75, -12.25]	2019
Akan E, 2021	44.76	146.9694	6	50	14.6969	6	0.1%	-5.24 [-123.42, 112.94]	2021
Ni, 2023	33.91	3	6	104.42	10.61	6	3.6%	-70.51 [-79.33, -61.69]	2023
**Subtotal (95% CI)**			**18**			**18**	**8.0%**	**-37.23 [-88.53, 14.07]**	
Heterogeneity: Tau² = 1567.23; Chi² = 151.64, df = 2 (P < 0.00001); I² = 99%									
Test for overall effect: Z = 1.42 (P = 0.15)									
**22.1.4 Adipose**									
Yea JH, 2021	32.2	1.2	5	51.9	1.8	5	4.3%	-19.70 [-21.60, -17.80]	2021
**Subtotal (95% CI)**			**5**			**5**	**4.3%**	**-19.70 [-21.60, -17.80]**	
Heterogeneity: Not applicable									
Test for overall effect: Z = 20.36 (P < 0.00001)									
**22.1.5 Others**									
Jiao YQ, 2011	25.87	17.8157	15	30.74	22.3471	15	2.8%	-4.87 [-19.33, 9.59]	2011
Pan, 2017	19.46	4.51	10	30.27	3.72	10	4.2%	-10.81 [-14.43, -7.19]	2017
Sheu JJ, 2020	44.8	1	6	61.57	3.53	6	4.2%	-16.77 [-19.71, -13.83]	2020
Liu L, 2021	22.5	6.6626	6	33.56	7.9853	6	3.7%	-11.06 [-19.38, -2.74]	2021
Rafiee Z, 2022	22.86	0.78	8	34	1.5	8	4.3%	-11.14 [-12.31, -9.97]	2022
**Subtotal (95% CI)**			**45**			**45**	**19.2%**	**-12.33 [-15.43, -9.23]**	
Heterogeneity: Tau² = 6.92; Chi² = 13.43, df = 4 (P = 0.009); I² = 70%									
Test for overall effect: Z = 7.80 (P < 0.00001)									
**Total (95% CI)**			**235**			**233**	**100.0%**	**-18.07 [-22.05, -14.09]**	
Heterogeneity: Tau² = 94.80; Chi² = 775.00, df = 28 (P < 0.00001); I² = 96%									
Test for overall effect: Z = 8.90 (P < 0.00001)									
Test for subgroup differences: Chi² = 17.08, df = 4 (P = 0.002), I² = 76.6%									

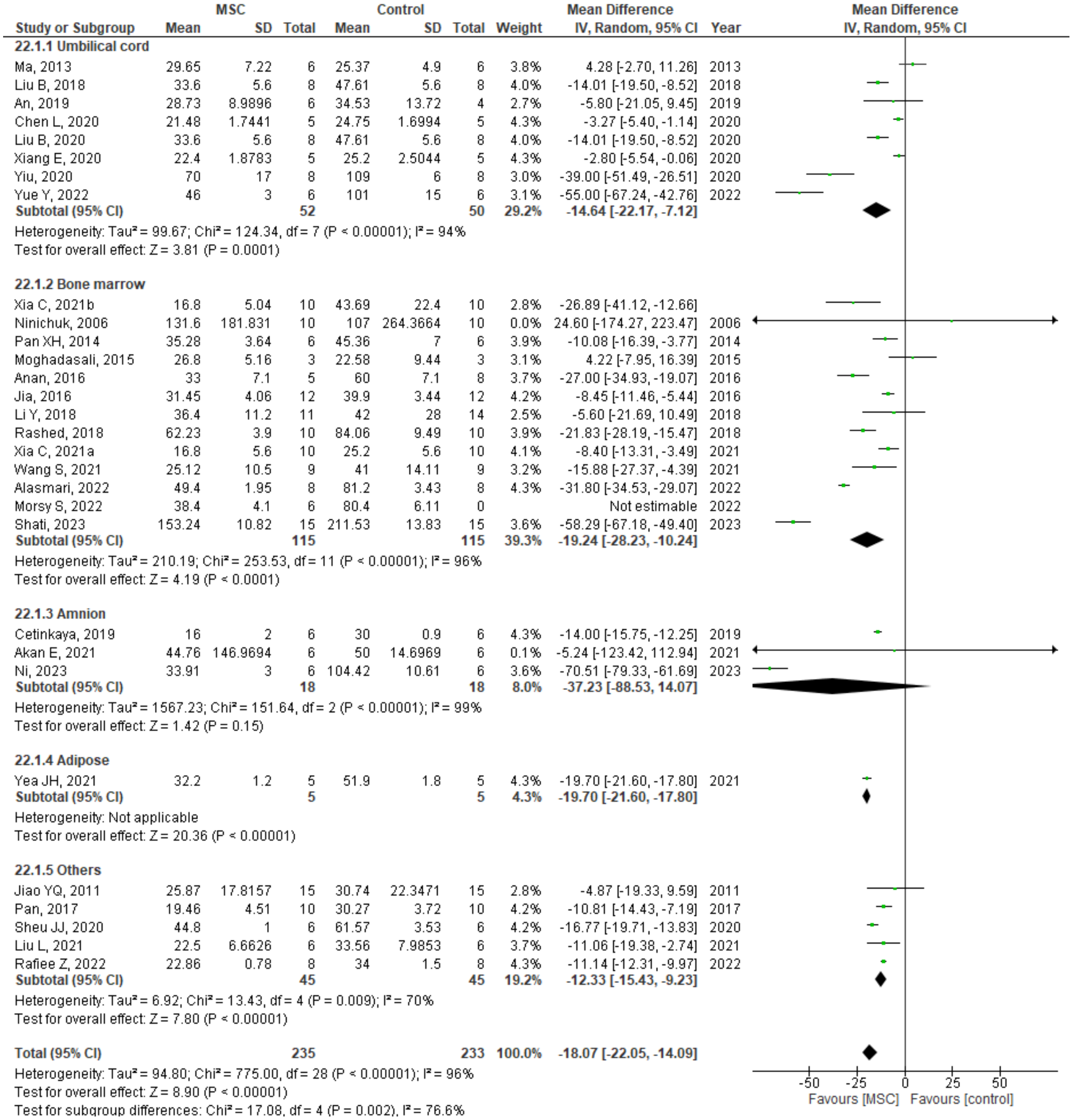

Figure S7. Forest plot of the effect of MSC treatment on BUN (mg/dL) in animal studies with subgroup analysis based on source of MSC (umbilical cord, bone marrow, amnion, adipose tissue, and others). Figure was generated using Review Manager (RevMan) version 5.4, The Cochrane Collaboration.

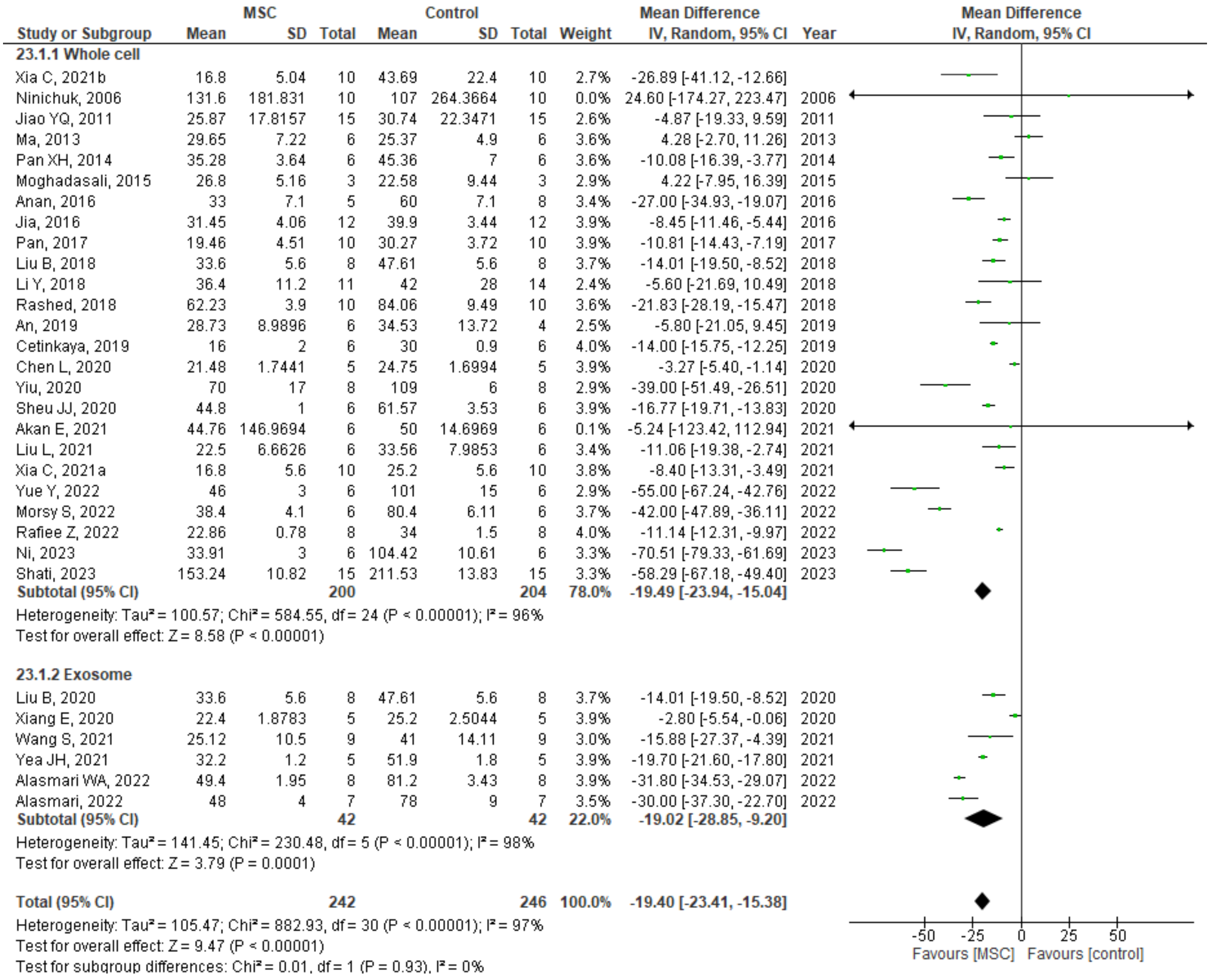

Figure S8. Forest plot of the effect of MSC treatment on BUN (mg/dL) in animal studies with subgroup analysis based on MSC-derived product (whole cell and exosome). Figure was generated using Review Manager (RevMan) version 5.4, The Cochrane Collaboration.

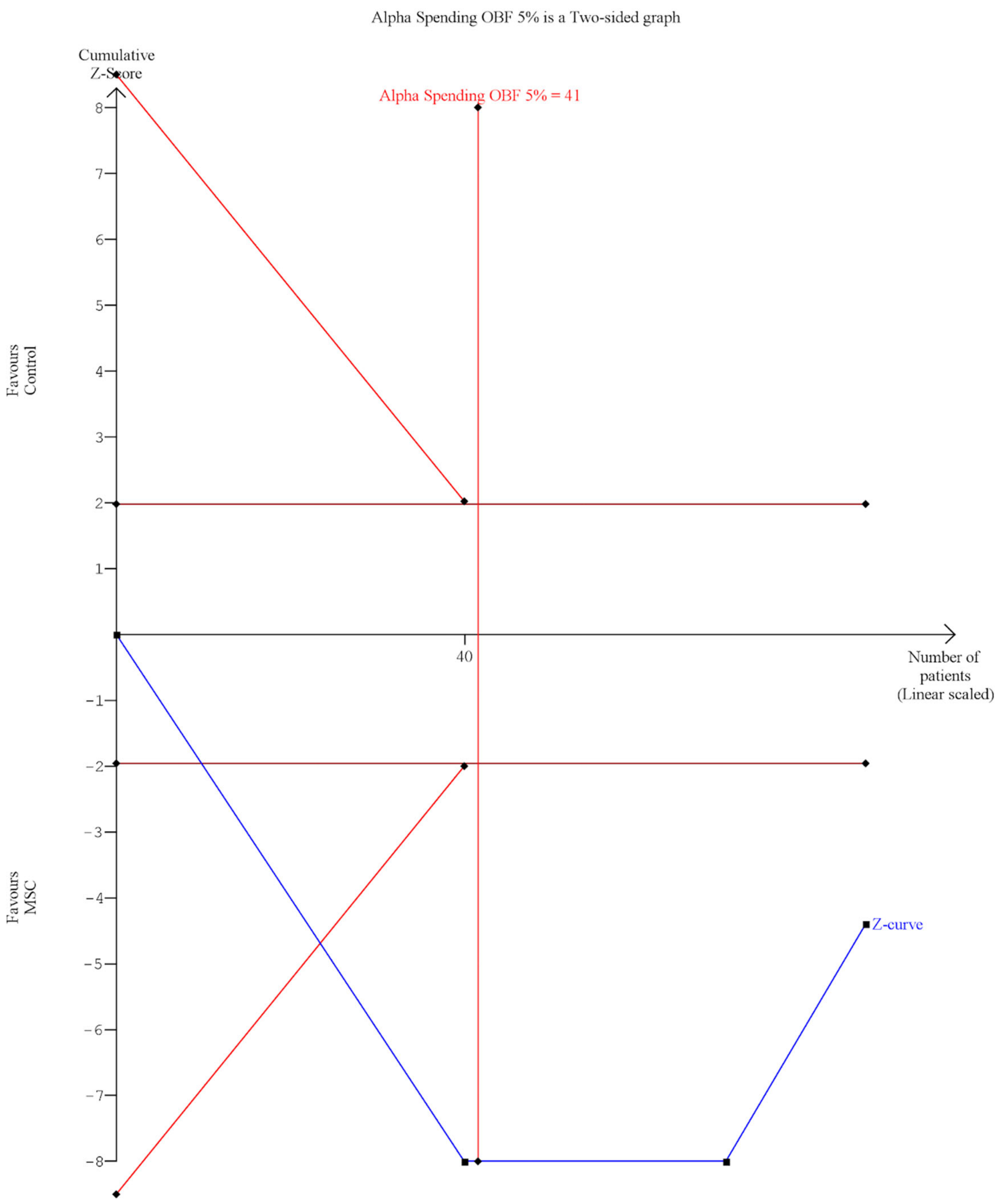

Figure S9. TSA Analysis for GFR in human RCTs. Using the specified parameters (alpha 5%, beta 20% [power 80%], diversity-adjusted required information size), the cumulative Z-curve crossed the LDOF monitoring boundary for benefit and reached the required information size. The TSA-adjusted confidence interval indicates a statistically significant improvement in GFR with MSC treatment.

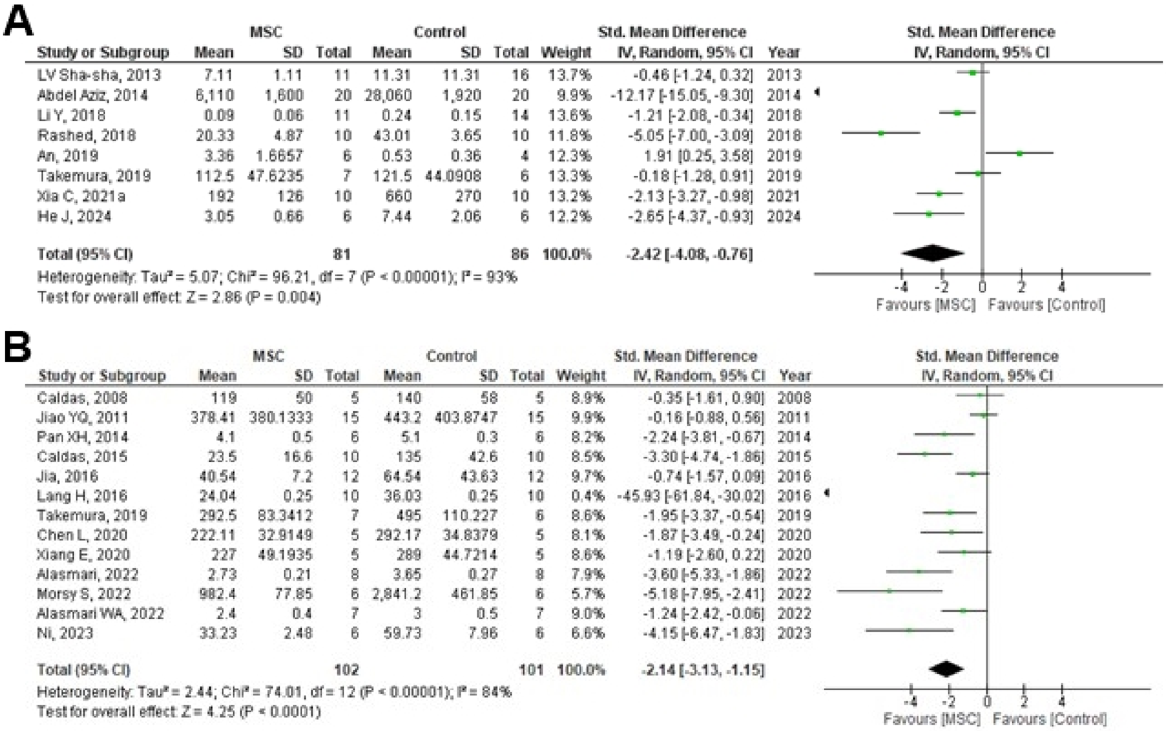

Figure S10. Forest plots of the effect of MSC treatment on (a) Albuminuria (mg/24h) in animal studies, (b) Proteinuria (mg/24h) in animal studies. Figure was generated using Review Manager (RevMan) version 5.4, The Cochrane Collaboration.

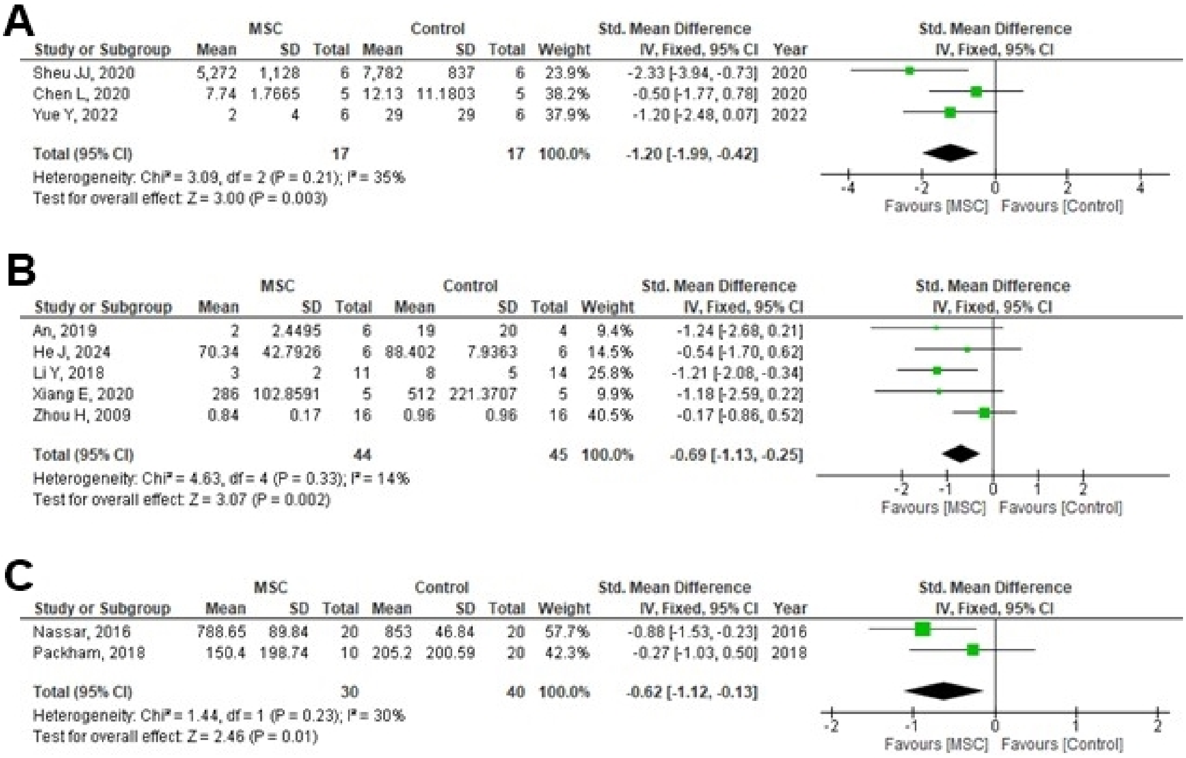

Figure S11. Forest plots of the effect of MSC treatment on (a) PCR in animal studies, (b) ACR in animal studies, (c) ACR in human RCTs. Figure was generated using Review Manager (RevMan) version 5.4, The Cochrane Collaboration.

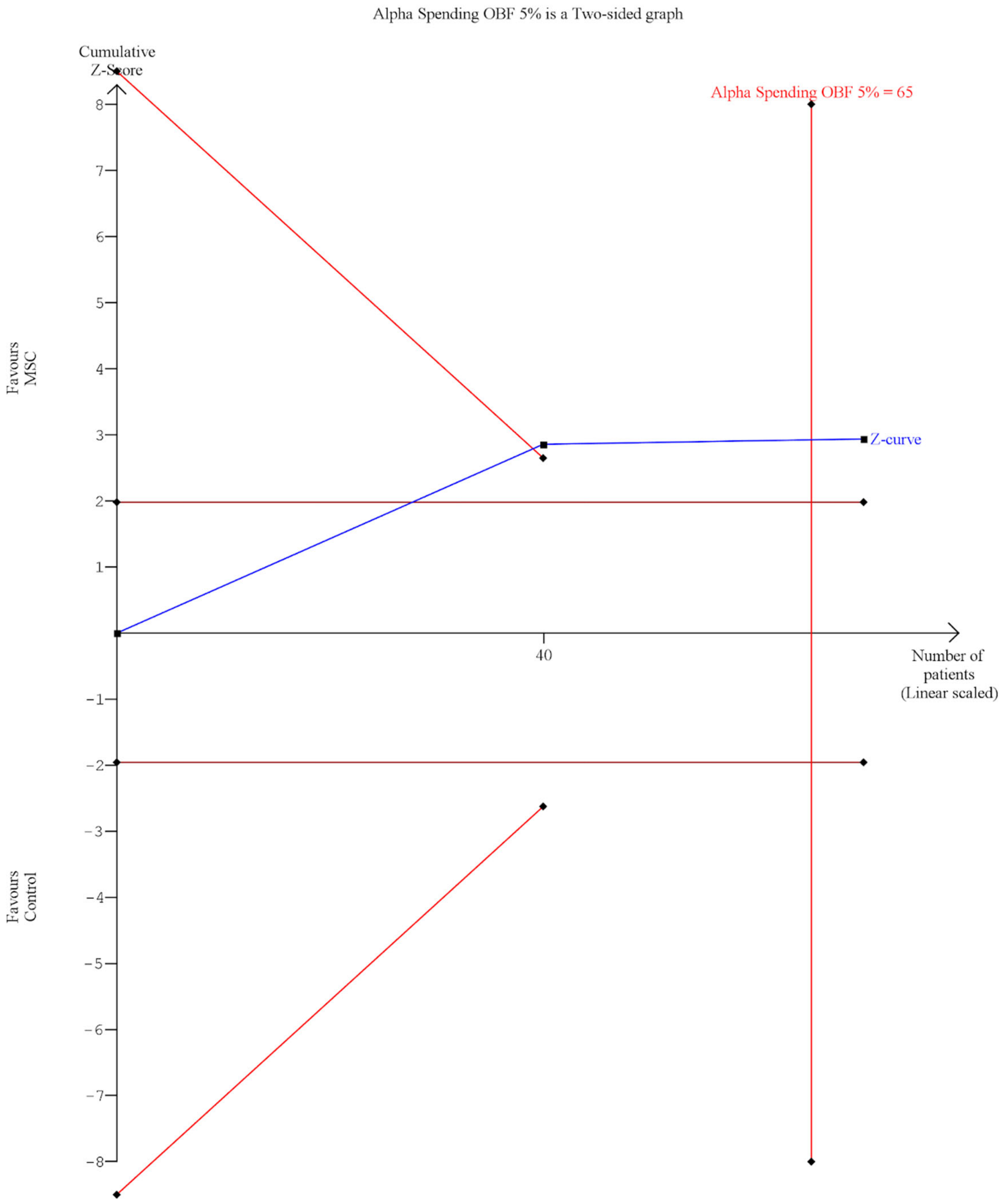

Figure S12. TSA Analysis for ACR in human RCTs. Using specified parameters (alpha 5%, beta 20% [power 80%], diversity-adjusted required information size), the cumulative Z-curve crossed the LDOF monitoring boundary for benefit and reached the required information size. The TSA-adjusted confidence interval confirms a significant reduction in ACR with MSC therapy.

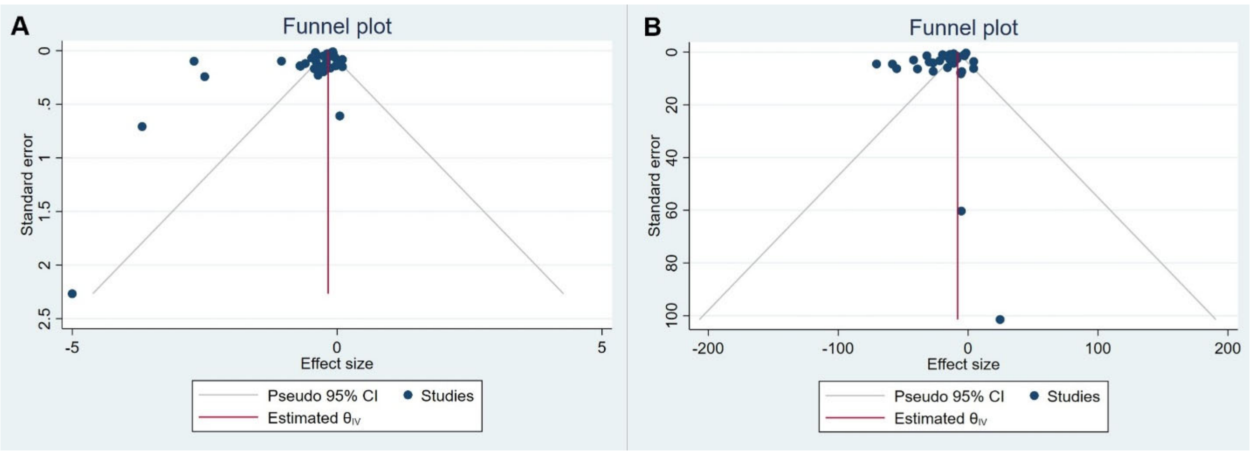

Figure S13. Publication bias assessment using funnel plots of (a) Scr outcome, showing asymmetry, with Egger's test indicating significant small-study effects (p = 0.0003), suggesting potential publication bias; (b) BUN outcome, appearing symmetrical, with Egger's test showing no significant small-study effects (p = 0.9424), suggesting no evidence of publication bias. The vertical red line represents the pooled effect estimate, and the diagonal lines represent the pseudo 95% confidence limits. Funnel plots were generated using Stata version 17 (StataCorp LLC, College Station, TX, USA).
